# Supplementary material for: miRNA-34a-5p is a novel biomarker and modulator of the radioresponse in esophageal adenocarcinoma
Source: Mol Ther Nucleic Acids. 2026 Aug 18;37(3):103064. doi: 10.1016/j.omtn.2026.103064 (PMC13570317; doi:10.1016/j.omtn.2026.103064)
Supplement: Supplementary file 1 [file mmc1.pdf]

OMTN, Volume *37*

Supplemental information

miRNA-34a-5p is a novel biomarker and modulator of the radioresponse in esophageal adenocarcinoma

Christina Cahill, David Hackett, Laura E. Kane, James J. Phelan, Jason McGrath, Maitiú Ó. Murchú, Aoife Cannon, Aisling B. Heeran, Aoife Kilgallon, Rebecca Lyons, Rebecca M. O'Brien, Wei-Lin Winnie Wang, Meghana S. Menon, Oana M. Deac, Antonia Tierney, Narayanasamy Ravi, Claire Donohoe, Dermot O'Toole, Cian Muldoon, Aoife Maguire, Joanne Lysaght, Maeve A. Lowery, John V. Reynolds, Stephen G. Maher, Jacintha O'Sullivan, and Niamh Lynam-Lennon

Table S1: Cox regression multivariate analysis of prognostic ability

Parameter	HR	CI	*p* value
miR-34a expression<br>(low vs. high)	0.257	0.077-0.856	0.027 *
TRG<br>(TRG 1-2 vs. 3-5)	0.285	0.061-1.341	0.112
Tumour Size<br>(small vs. large)	0.492	0.173-1.397	0.183
Tumour differentiation<br>(poor + poor/moderate vs. moderate/well)	0.424	0.139-1.297	0.132
Pathological T stage<br>(T0-1 vs. T2-3)	3.892	1.069-14.171	0.039 *
Pathological N stage<br>(N0 vs. N1-2)	2.157	0.632-7.359	0.219

Abbreviations; HR, hazard ratio; CI, confidence interval; TRG, tumour regression grade; T stage, tumour stage; N stage, lymph node stage.

Table S2: Optimised clonogenic seeding densities for assessment of the radiosensitivity of OAC cells

Cell line	Treatment	Cells seeded per well (6-well plate)	Days incubated
OE19	0 Gy<br>2 Gy	1.5×10^3<br>3×10^3	~ 10
OE33	0 Gy<br>2 Gy	1.5×10^3<br>3×10^3	~ 7
OE33 P/R	0 Gy<br>2 Gy	1.5×10^3<br>3×10^3	~ 7
Flo-1^{Par}	0 Gy<br>2 Gy<br>4 Gy<br>6 Gy	1×10^3<br>2×10^3<br>4×10^3<br>6×10^3	~ 10
Flo-1^{LM}	0 Gy<br>2 Gy<br>4 Gy<br>6 Gy	1.5×10^3<br>3×10^3<br>6×10^3<br>1.2×10^4	~ 10

Abbreviations; Gy, Gray
