# Supplementary material for: miRNA-34a-5p is a novel biomarker and modulator of the radioresponse in esophageal adenocarcinoma
Source: Mol Ther Nucleic Acids. 2026 Aug 18;37(3):103064. doi: 10.1016/j.omtn.2026.103064 (PMC13570317; doi:10.1016/j.omtn.2026.103064)
Supplement: Supplementary file 2 [file mmc2.pdf]

Molecular Therapy
Nucleic Acids
Original Article

miRNA-34a-5p is a novel biomarker and modulator of the radioresponse in esophageal adenocarcinoma

Christina Cahill,[1] David Hackett,[1] Laura E. Kane,[1] James J. Phelan,[1] Jason McGrath,[1,2] Maitiú Ó. Murchú,[1,3] Aoife Cannon,[1] Aisling B. Heeran,[1,3] Aoife Kilgallon,[1] Rebecca Lyons,[1] Rebecca M. O'Brien,[1] Wei-Lin Winnie Wang,[4] Meghana S. Menon,[1] Oana M. Deac,[1,5] Antonia Tierney,[5] Narayanasamy Ravi,[1] Claire Donohoe,[1] Dermot O'Toole,[6] Cian Muldoon,[7] Aoife Maguire,[7] Joanne Lysaght,[1] Maeve A. Lowery,[1] John V. Reynolds,[1] Stephen G. Maher,[1] Jacintha O'Sullivan,[1] and Niamh Lynam-Lennon[1,8,9]

[1]Department of Surgery, School of Medicine, Trinity Translational Medicine Institute and Trinity St. James's Cancer Institute, Trinity College Dublin, Dublin 8, Dublin, Ireland; [2]Department of Surgery, RCSI University of Medicine and Health Sciences, Dublin 2, Dublin, Ireland; [3]School of Pharmacy and Biomolecular Sciences, RCSI University of Medicine and Health Sciences, Dublin 2, Dublin, Ireland; [4]miR Scientific LLC, Rensselaer, NY 12144, USA; [5]Department of Medical Oncology, St James's Hospital, Dublin 8, Dublin, Ireland; [6]Department of Clinical Medicine, Trinity Translational Medicine Institute and Trinity St. James's Cancer Institute, St. James's Hospital, Trinity College Dublin, Dublin 8, Dublin, Ireland; [7]Department of Pathology, St James's Hospital, Dublin 8, Dublin, Ireland; [8]Department of Biology, Kathleen Lonsdale Institute for Human Health Research, National University of Ireland Maynooth, Maynooth, W23 YCW7, Kildare, Ireland

Poor pathological response to neoadjuvant chemoradiotherapy (neo-CRT) remains a major clinical challenge in esophageal adenocarcinoma (EAC), contributing to treatment resistance and poor patient outcomes. Identification of novel predictive biomarkers of treatment resistance and therapeutic targets to enhance treatment response are crucial to improving patient outcomes. In this study, we investigated the role of miR-34a-5p in EAC treatment response using both cell line models and clinical tumor samples. We observed reduced miR-34a-5p expression across multiple cell line models of radioresistant EAC. Overexpression of miR-34a-5p enhanced radiosensitivity, impaired DNA damage repair, and altered cell cycle following irradiation, accompanied by changes in the expression of DNA damage response and cell cycle-related genes. Consistent with these findings, analysis of pre-treatment tumor biopsies from EAC patients demonstrated that reduced miR-34a-5p expression was associated with poor response to neo-CRT, advanced pathological T stage, and worse clinical outcomes. This study demonstrates for the first time, a role for miR-34a-5p as a potential biomarker of treatment response and an independent predictor of survival outcomes in EAC patients. Furthermore, we identify miR-34a-5p as a radiosensitizer in EAC, highlighting a novel therapeutic approach to enhance the efficacy of radiation therapy in EAC.

INTRODUCTION

Cancer of the esophagus represents a significant global health burden, accounting for approximately 1 in every 20 cancer deaths worldwide.[1] In recent decades, there has been an alarming growth in the incidence of esophageal adenocarcinoma (EAC), with increases linked to the rising prevalence of gastro-esophageal reflux disease (GERD) and obesity.[2] Despite recent advances in diagnosis and surveillance, survival outcomes remain poor, and the 5-year survival rate for EAC patients globally is approximately 20%.[3] Of particular concern is the rise in the incidence of early onset EAC in patients <50 y, with these patients often presenting with advanced tumors, which are more aggressive and refractory to treatment, resulting in poorer survival outcomes.[4]

To combat poor survival rates, a multimodal approach to curative treatment has been adopted for EAC patients. While perioperative chemotherapy regimens such as FLOT (fluorouracil, leucovorin, oxaliplatin, and docetaxel) are increasingly becoming the standard of care, neoadjuvant chemoradiation therapy (neo-CRT) followed by surgery remains a commonly utilized treatment strategy, which has shown long-term survival benefits, when compared to surgery alone.[5] The tumor's pathological response to neo-CRT is assessed following surgery, and achievement of a pathological complete response (pCR), characterized by no viable tumor cells, is associated with improved survival outcomes for patients.[6] However, unfortunately less than 30% of EAC patients achieve a pCR following neo-CRT.[7] Consequently, the remaining ∼70% of patients who demonstrate treatment resistance are subject to therapy-associated toxicities with no apparent therapeutic benefit and, as CRT is given

Received 18 March 2026; accepted 13 August 2026;
https://doi.org/10.1016/j.omtn.2026.103064.

[9]Senior author

Correspondence: Niamh Lynam-Lennon, Department of Biology, Kathleen Lonsdale Institute for Human Health Research, National University of Ireland Maynooth, Maynooth, W23 YCW7, Kildare, Ireland
E-mail: niamh.lynamlennon@mu.ie

© 2026 The Author(s).
Published by Elsevier Inc. on behalf of The American Society of Gene and Cell Therapy.
This is an open access article under the CC BY license (http://creativecommons.org/licenses/by/4.0/).

neoadjuvantly, the delay to surgery may worsen prognosis. Thus, accurate prediction of those patients who are likely to be sensitive or resistant to treatment is crucial for improving treatment stratification, quality of life, and survival outcomes for EAC patients. Currently, standard clinicopathological factors do not predict treatment response, and no predictive biomarkers are routinely used in clinical practice.

Patients with tumors of similar characteristics can have vastly different responses to treatment, suggesting that differences in tumor sensitivity are due to subtle differences in the tumor molecular environment. This suggests that regulatory molecules, such as microRNAs (miRNAs) may play an important role in the response to neo-CRT. miRNAs are small (18–24 nucleotides) single-stranded, non-protein coding RNA molecules that regulate gene expression by either degrading the target mRNA or repressing translation, effectively downregulating target genes.[8] Importantly, a single miRNA can target a number of genes simultaneously.[9] Thus, miRNAs play an essential role as master gene regulators, often acting as either oncogenes or tumor suppressor genes. miR-34a is a member of the miR-34 family, which also includes miR-34b/c. All three family members are directly regulated by the tumor suppressor p53, which binds to the miR-34 gene promoter, activating transcription.[10] Moreover, miR-34a is a known tumor suppressor, targeting and downregulating a number of oncogenes, promoting cell-cycle arrest, and enhancing apoptosis.[11] miR-34a is located on chromosome 1p36.22 and is often decreased in cancer due to deletion of the 1p36 locus,[12] epigenetic silencing,[13] or regulation by oncogenes, such as Myc.[14] miR-34a downregulation is associated with treatment resistance in various cancer types, including cisplatin resistant gastric cancer[15] and radioresistant colon cancer.[16] However, its role in the treatment response of EAC is largely unknown.

The cellular response to radiation therapy is governed by factors such as the extent of DNA damage induced, the efficiency of DNA repair mechanisms, the balance between pro-apoptotic and anti-apoptotic signals, and the activation of cell-cycle checkpoints.[17] Alterations in these pathways are frequently associated with radioresistance.[18] miR-34a has been demonstrated to contribute to treatment response in a number of cancer types via regulation of key pathways involved in the response to cytotoxic treatment, such as the DNA damage response,[19] cell cycle,[20] and apoptosis.[21] Thus, evidence indicates that miR-34a may serve not only as a biomarker to predict treatment response but also as a promising therapeutic target to enhance treatment efficacy.

Our group has previously demonstrated that miRNAs play a role in the response of EAC to both X-ray radiation and chemotherapy,[22–24] highlighting their potential as both predictive biomarkers and as novel therapeutic targets to improve treatment response in EAC. However, the role of miR-34a in radioresistant EAC is unknown. This study utilizes *in vitro* models of both inherent and acquired radioresistance in EAC. Importantly, we demonstrate that decreased miR-34a-5p expression is associated with radioresistance in EAC cell lines. This is supported in EAC patients, whereby decreased miR-34a-5p expression in pre-treatment EAC tumor biopsies is associated with resistance to neo-CRT, advanced pathological T stage, and poor prognosis. This study also demonstrates for the first time that miR-34a-5p functionally modulates radiosensitivity, acting as a novel radiosensitizer via impairment of DNA damage repair, modulation of cell-cycle distribution, and regulation of key genes involved in the cell cycle and DNA damage response. We identify cyclin E2 (CCNE2) as a potential direct target of miR-34a-5p, suggesting that loss of miR-34a-5p in EAC may promote therapeutic resistance and unfavorable prognosis through CCNE2 upregulation. Collectively, these findings support a role for miR-34a as a biomarker predicting response to neo-CRT for patients with EAC and highlight the potential of miR-34a delivery as a novel therapeutic strategy to enhance the radioresponse in EAC.

RESULTS

Radioresistant EAC cell lines have decreased miR-34a-5p expression

The radiosensitivity of a panel of EAC cells lines to a clinically relevant dose of 2 Gray (Gy) X-ray radiation was assessed by clonogenic assay to identify *in vitro* models of radioresistant EAC. OE19 cells, previously demonstrated to exhibit inherent radioresistance, compared to OE33 cells,[24] and the isogenic OE33 P (parental) and OE33 R (radioresistant) cell lines previously developed by our group[25] were utilized. Additionally, FLO-1 parental (FLO-1^{Par}) and FLO-1^{LM}, a model of primary EAC and liver metastasis[26] were also assessed.

Supporting previous data, OE33 R cells were significantly more radioresistant to 2 Gy, when compared to age- and passage-matched OE33 P cells (p = 0.0026) (Figure 1A). Similarly, OE19 cells were significantly more radioresistant to 2 Gy, when compared to age- and passage-matched OE33 cells (p = 0.0016) (Figure 1B). Interestingly, FLO-1^{LM} cells were significantly more radioresistant to 2 Gy than FLO-1^{Par} cells (p = 0.0206) (Figure 1C). These data demonstrate for the first time that FLO-1^{LM} cells represent not only a model of EAC progression but also a model of inherent radioresistance and support OE19 cells and OE33 R cells as robust models of inherent and acquired radioresistant EAC, respectively.

miR-34a-5p expression was significantly decreased in radioresistant OE33 R cells, when compared to radiosensitive OE33 P cells (p = 0.0453) (Figure 1D), in radioresistant OE19 cells, when compared to radiosensitive OE33 cells (p = 0.0462) (Figure 1E), and in radioresistant FLO-1^{LM} cells, when compared to radiosensitive FLO-1^{Par} cells (p = 0.0013) (Figure 1F). While irradiation did not significantly alter miR-34a-5p expression in OE33 P, OE33 R (Figure 1D), OE33, OE19 (Figure 1E), or FLO-1^{LM} (Figure 1F) cells, miR-34a-5p expression was significantly increased in FLO-1^{Par} cells following 2 Gy, when compared to unirradiated controls (Figure 1F). Across all

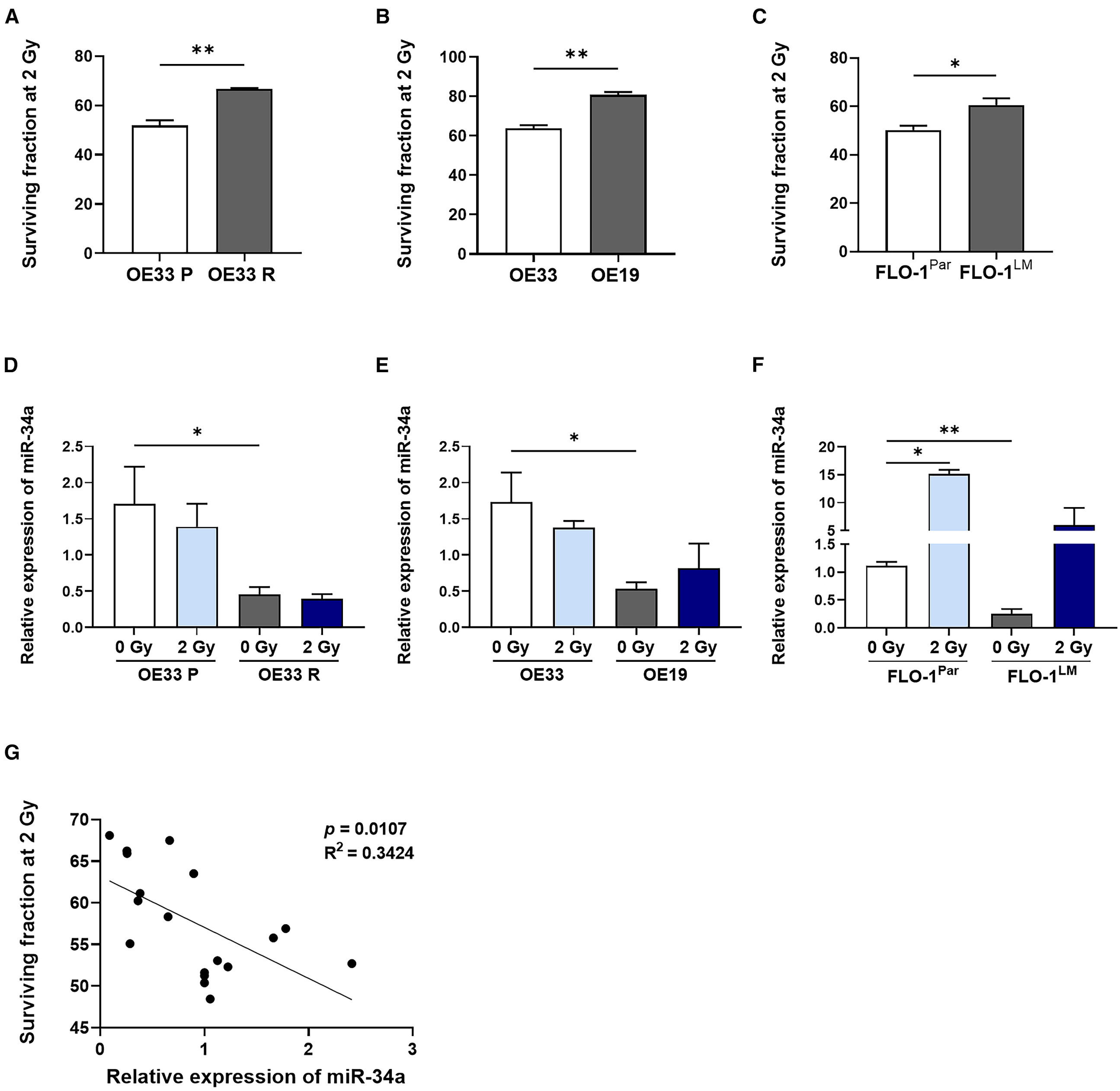

Figure 1. miR-34a-5p expression is significantly decreased in radioresistant EAC cells

The radiosensitivity of EAC cell lines following irradiation with a clinically relevant dose of 2 Gy X-ray radiation was assessed by clonogenic assay. (A–C) Surviving fraction following treatment with 2 Gy X-ray radiation in (A) OE33 P and OE33 R cells (n = 3), (B) OE33 and OE19 cells (n = 3), and (C) FLO-1^{Par} and FLO-1^{LM} cells (n = 3). miR-34a-5p expression was assessed basally and at 24 h post-irradiation with 2 Gy X-ray radiation by qPCR. Controls were mock irradiated. (D–F) Expression of miR-34a-5p in (D) OE33 P and OE33 R cells (n = 5), (E) OE33 and OE19 cells (n = 3), and (F) FLO-1^{Par} and FLO-1^{LM} cells (n = 3). Data are presented as mean ± SEM. Statistical analysis was performed using paired and unpaired two-tailed Student's t tests. (G) miR-34a-5p expression was correlated with surviving fraction at 2 Gy in EAC cell lines (n = 18). Statistical analysis was performed by calculating the Pearson correlation coefficient. *$p < 0.05$, **$p < 0.01$.

in vitro models, relative miR-34a-5p expression negatively correlated with surviving fraction at 2 Gy ($p = 0.0107$, $R^2 = 0.0107$) (Figure 1G), supporting a relationship between decreased miR-34a-5p expression and radioresistance *in vitro* in EAC.

miR-34a-5p functionally modulates radiosensitivity in radioresistant OE33 R EAC cells

The functional role of miR-34a-5p in modulating radiosensitivity in EAC was investigated by stable overexpression of miR-34a in

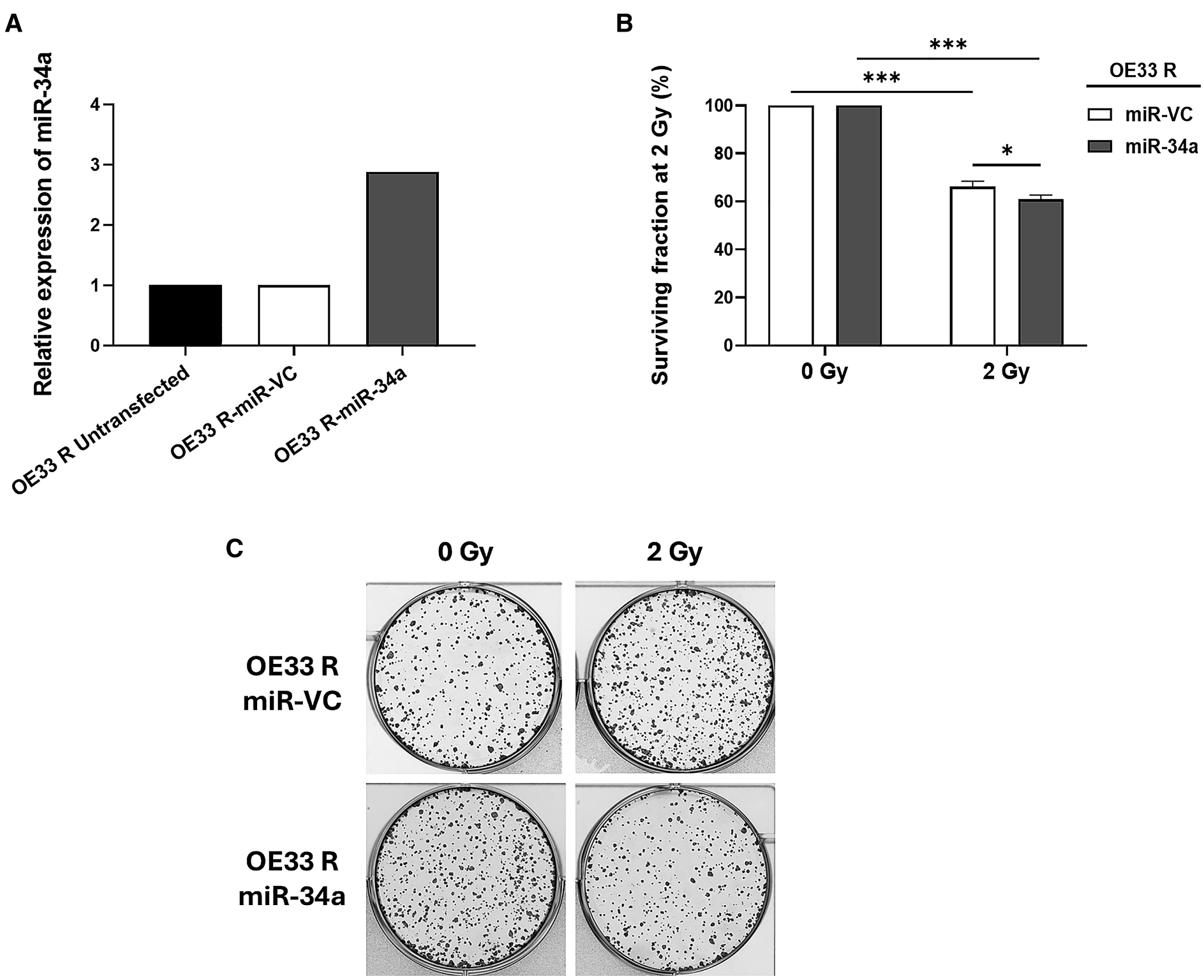

Figure 2. miR-34a radiosensitizes radioresistant OE33 R EAC cells

OE33 R cells were stably transfected with miR-34a plasmid (5 μg) or vector control (miR-VC). miR-34a-5p overexpression was confirmed by qPCR. (A) miR-34a-5p expression in OE33 R untransfected, miR-VC transfected, and miR-34a transfected cells. Data are presented as mean relative expression from a single experiment. (B) The radiosensitivity of OE33 R-miR-VC and OE33 R-miR-34a cells following 2 Gy X-ray radiation was assessed by clonogenic assay ($n = 3$). (C) Representative images of colonies formed following the clonogenic period. Controls were mock irradiated. Data are presented as mean surviving fraction ±SEM. Statistical analysis was performed by paired and unpaired two-tailed Student's t test. *$p < 0.05$, *$p < 0.001$.

radioresistant OE33 R cells. OE33 R cells were transfected with a pCMV-miR-34a construct expressing the precursor miR-34a, enabling endogenous processing into mature miRNA strands. The effect of miR-34a overexpression on radiosensitivity at a clinically relevant dose of 2 Gy was assessed by clonogenic assay.

miR-34a-5p overexpression was confirmed by quantitative real-time PCR (qPCR) (Figure 2A). Irradiation with 2 Gy significantly reduced the surviving fraction of both OE33 R-miR-34a cells (p = 0.0003) and cells transfected with a scrambled control (OE33 R-miR-VC) (p = 0.0008), compared to unirradiated controls. However, interestingly, OE33 R-miR-34a cells were significantly (p = 0.0372) more sensitive to 2 Gy, when compared to OE33 R-miR-VC cells (mean relative expression ±SEM; OE33 R-miR-VC 66.15 ± 1.349, OE33 R-miR-34a 60.94 ± 1.027) (Figures 2B and 2C). These data demonstrate for the first time that miR-34a-5p plays a functional role in modulating the response to radiation in radioresistant OE33 R cells, enhancing radiosensitivity.

miR-34a-5p impairs DNA damage repair following irradiation in radioresistant EAC cells

The role of miR-34a-5p in radiation-induced DNA damage was investigated in OE33 R-miR-VC and OE33 R-miR-34a cells by measurement of γH_2AX, a marker for DNA double-strand breaks (DSBs),[27] by flow cytometry at 20 min post-irradiation with 2 Gy. A significant induction of DNA damage was observed in both OE33 R-miR-VC cells and OE33 R-miR-34a cells, relative to unirradiated controls (Figure 3A). However, similar levels of radiation-induced DNA damage were observed between OE33 R-miR-VC and OE33 R-miR-34a cells (Figure 3A).

Loss of phosphorylation of H_2AX is associated with the repair of DSBs.[27] Thus, the role of miR-34a-5p in DNA damage repair

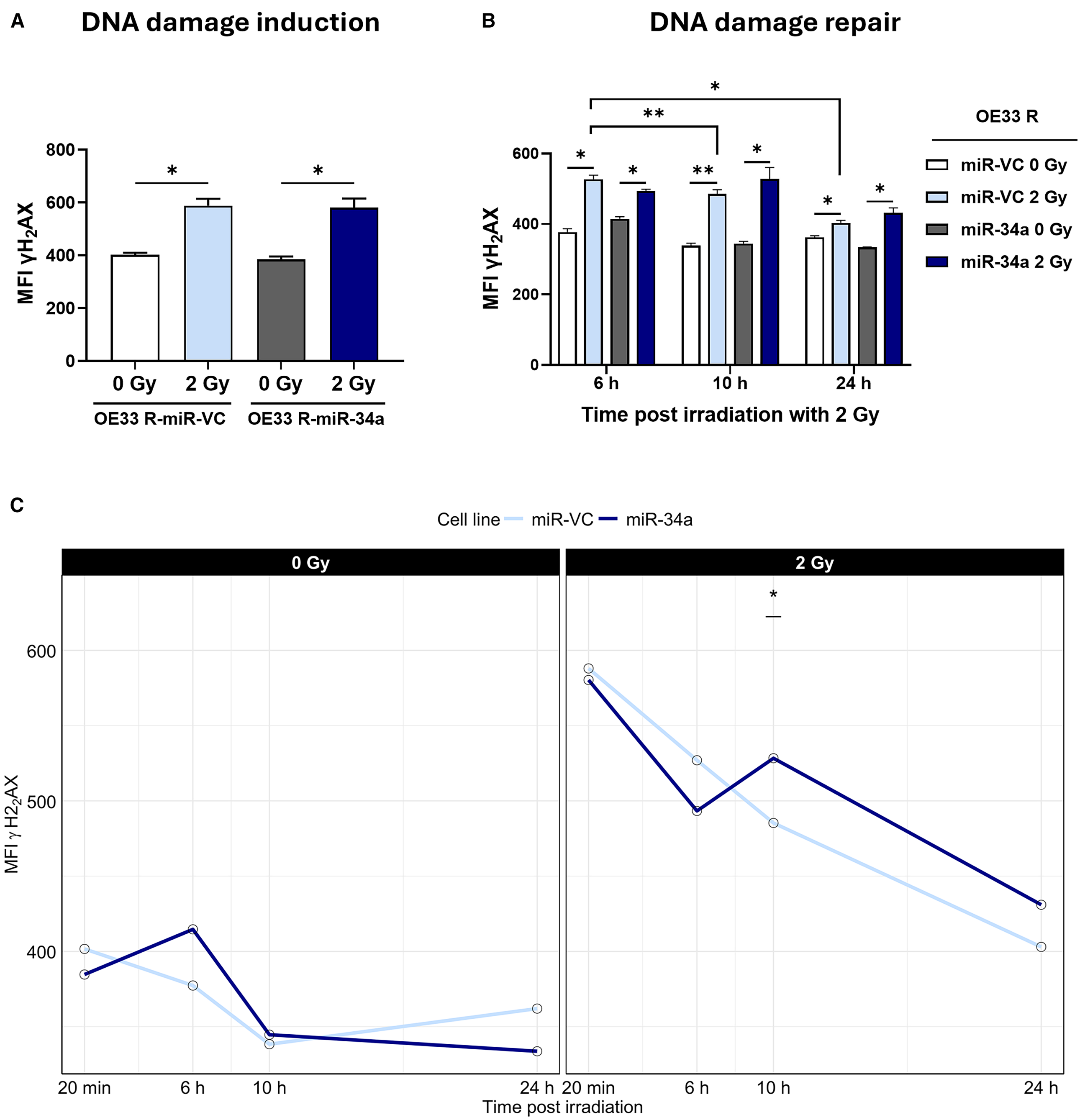

Figure 3. miR-34a overexpression impairs DNA damage repair in radioresistant OE33 R EAC cells

DNA damage was assessed in OE33 R-miR-VC and OE33 R-miR-34a cells by measurement of γH_2AX by flow cytometry (*n* = 3). (A) DNA damage induction was investigated by assessment of MFI of γH_2AX basally and at 20 min following 2 Gy X-ray radiation. Data are presented as mean ± SEM. Statistical analysis was performed by paired and unpaired two-tailed Student's *t* test. (B) DNA damage repair was investigated by assessment of MFI of γH_2AX at 6 h, 10 h, and 24 h following 2 Gy X-ray radiation. Data are presented as mean ± SEM. Statistical analysis was performed by two-way ANOVA and Tukey's post-hoc test. *$p < 0.05$, **$p < 0.01$. (C) Lines show model-predicted means from a linear mixed-effects model (cell line × dose × time, random intercept for replicate). Asterisk indicates $p < 0.05$ for the miR-34a vs. miR-VC contrast at 10 h after 2 Gy (mixed-effects post-hoc comparison).

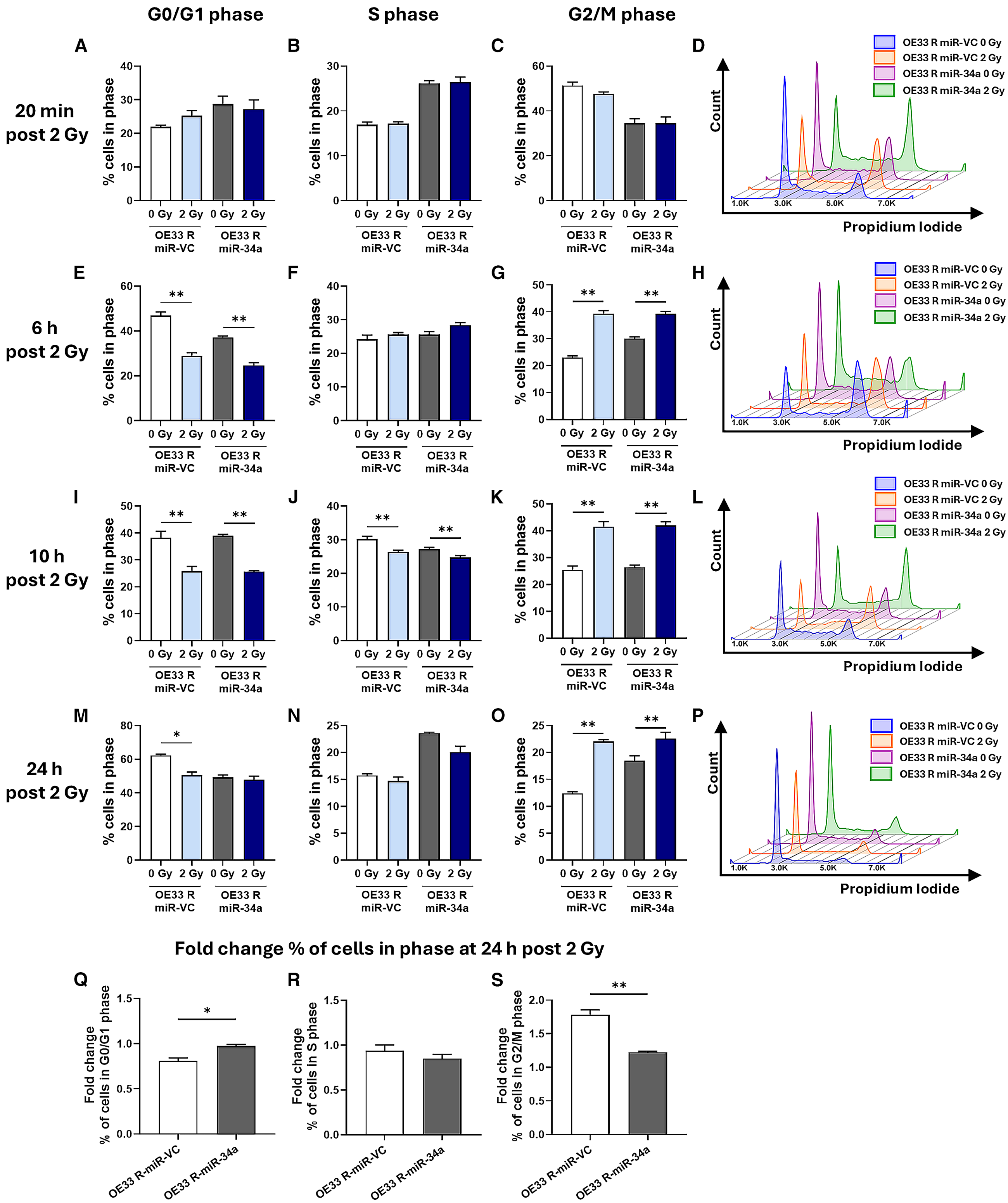

(legend on next page)

following irradiation was investigated by measurement of γH_2AX by flow cytometry at 6, 10, and 24 h post-irradiation with 2 Gy. When compared to unirradiated controls, a significant induction of DNA damage was maintained at 6, 10, and 24 h post-irradiation with 2 Gy in OE33 R-miR-VC cells and in OE33 R-miR-34a cells (Figure 3B). However, following irradiation with 2 Gy in OE33 R-miR-VC cells, there was a significant (p = 0.0088) decrease in γH_2AX levels at 10 h, when compared to 6 h post-irradiation and a further significant (p = 0.0485) decrease in γH_2AX levels at 24 h, when compared to 10 h post-irradiation (Figure 3B), suggesting repair of radiation-induced DSBs. In contrast, no significant decrease in γH_2AX levels was observed in OE33 R-miR-34a cells at these time points. Linear mixed-effects modeling identified a significant cell line × dose × time interaction ($F(3,30) = 3.58$, $p = 0.025$), indicating that miR-34a-5p subtly modulates the dose-dependent γH_2AX time course. Post-hoc contrasts revealed a modest but significant increase in γH_2AX in OE33 R-miR-34a cells, compared to OE33 R-miR-VC cells at 10 h after 2 Gy (estimate +43 a.u., 95% confidence interval [CI] 0.3–85.7, p = 0.049), while differences at other time points and doses were not statistically significant (Figure 3C). This suggests that miR-34a-5p overexpression impairs DNA damage repair in OE33 R cells following 2 Gy radiation, which may contribute to its radiosensitizing effect.

miR-34a-5p alters cell-cycle distribution following irradiation in radioresistant EAC cells

As cell-cycle regulation is tightly linked to the DNA damage response, the effect of miR-34a-5p overexpression on cell-cycle distribution following 2 Gy X-ray irradiation was investigated by PI staining and flow cytometry.

At 20 min post-irradiation with 2 Gy, there were no alterations in the percentage of OE33 R-miR-VC or OE33 R-miR-34a cells in G0/G1 phase, S phase, or G2/M phase, when compared to unirradiated controls (Figures 4A–4D). At 6 h post-irradiation (Figures 4E–4H), no alterations in the percentage of OE33 R-miR-34a or OE33 R-miR-VC cells in S phase were observed (Figure 4F). However, the percentage of OE33 R-miR-VC and OE33 R-miR-34a cells in G0/G1 phase was decreased (Figure 4E), accompanied by an increase in the percentage of cells in G2/M phase (Figure 4G). At 10 h post-irradiation (Figures 4I–4L), there remained a significantly decreased percentage of both OE33 R-miR-VC and OE33 R-miR-34a cells in G0/G1 phase (Figure 4I), accompanied by an increase in the percentage of cells in G2/M phase (Figure 4K). However, at this 10 h time point, a slight but significant decrease in the percentage of both OE33 R-miR-VC and OE33 R-miR-34a cells in S phase was also observed (Figure 4J). At 24 h post-irradiation (Figures 4M–4P), there were no alterations in the percentage of OE33 R-miR-VC or OE33 R-miR-34a cells in S phase (Figure 4N) but there remained a significant increase in the percentage of both OE33 R-miR-VC and OE33 R-miR-34a cells in G2/M phase, when compared to unirradiated controls (Figure 4O). Additionally, a significant (p = 0.0272) decrease was observed in the percentage of OE33 R-miR-VC cells in G0/G1 phase, compared to unirradiated controls. However, interestingly, this decrease was not observed in OE33 R-miR-34a cells (Figure 4M).

Due to the cell-cycle distribution differences observed between OE33 R-miR-VC and OE33 R-miR-34a cells at 24 h following radiation, fold change was assessed to further evaluate these changes. Investigating the fold change in percentage of cells in each phase at 24 h post-2 Gy irradiation revealed a similar proportion of OE33 R-miR-VC and OE33 R-miR-34a cells in S phase (Figure 4R) but a significant (p = 0.0117) increase in the proportion of OE33 R-miR-34a cells in G0/G1 phase (Figure 4Q), accompanied by a significant (p = 0.0016) decrease in the proportion of OE33 R-miR-34a cells in G2/M phase (Figure 4S), when compared to OE33 R-miR-VC cells. This suggests that miR-34a-5p overexpression may promote G1 arrest and inhibit progression into the G2/M phase following irradiation, potentially enhancing the DNA damage response and contributing to the radiosensitization of OE33 R cells.

miR-34a-5p alters expression of cell cycle- and DNA repair-related genes in radioresistant EAC cells

Having identified alterations in cell cycle and DNA damage repair as potential mechanisms underlying the miR-34a-mediated radiosensitization of EAC cells, identification of putative miR-34a-regulated mRNA targets in OE33 R cells was performed. Expression of 44 cell cycle-related genes was assessed in OE33 R-miR-VC and OE33 R-miR-34a cells using TaqMan PCR arrays. Of the 44 genes investigated, 43 were detected in all samples. A total of 27 genes were significantly altered in OE33 R-miR-34a cells, when compared to OE33 R-miR-VC cells (Figures 5A–5F). Of these, 19 genes were significantly increased while 8 were significantly decreased in OE33 R-miR-34a cells, when compared to OE33 R-miR-VC cells.

The expression of three cyclin genes, CCNA2, CCND3, and CCNE2 were significantly decreased and expression of CCNB1, CCNB2, and CCNH were significantly increased in OE33 R-miR-34a cells, when compared to OE33 R-miR-VC cells (Figure 5A). The expression of CDK genes CDK2, CDK4, and CDK7 were significantly increased in OE33 R-miR-34a cells, compared to OE33 R-miR-VC cells

Figure 4. miR-34a overexpression alters cell-cycle distribution post-irradiation with 2 Gy in radioresistant OE33 R EAC cells

(A–C, E–G, I–K, and M–O) OE33 R-miR-VC and OE33 R-miR-34a cells were irradiated with 2 Gy (n = 3). Controls were mock irradiated. At (A–C) 20 min, (E–G) 6 h, (I–K) 10 h, and (M–O) 24 h post-irradiation with 2 Gy, cells were fixed and stained with PI to assess cell cycle distribution. (D, H, L, and P) Representative histograms (one repeat) demonstrate the number of cells in each stage of the cell cycle at (D) 20 min, (H) 6 h, (L) 10 h, and (P) 24 h post-irradiation with 2 Gy. (Q, R, and S) At 24 h post-irradiation with 2 Gy, the fold change in % of cells in (Q) G0/G1, (R), S, and (S) G2/M phase were assessed relative to unirradiated controls. Data are presented as mean ± SEM. Statistical analysis was performed by paired and unpaired two-tailed Student's t test. *$p < 0.05$, **$p < 0.01$.

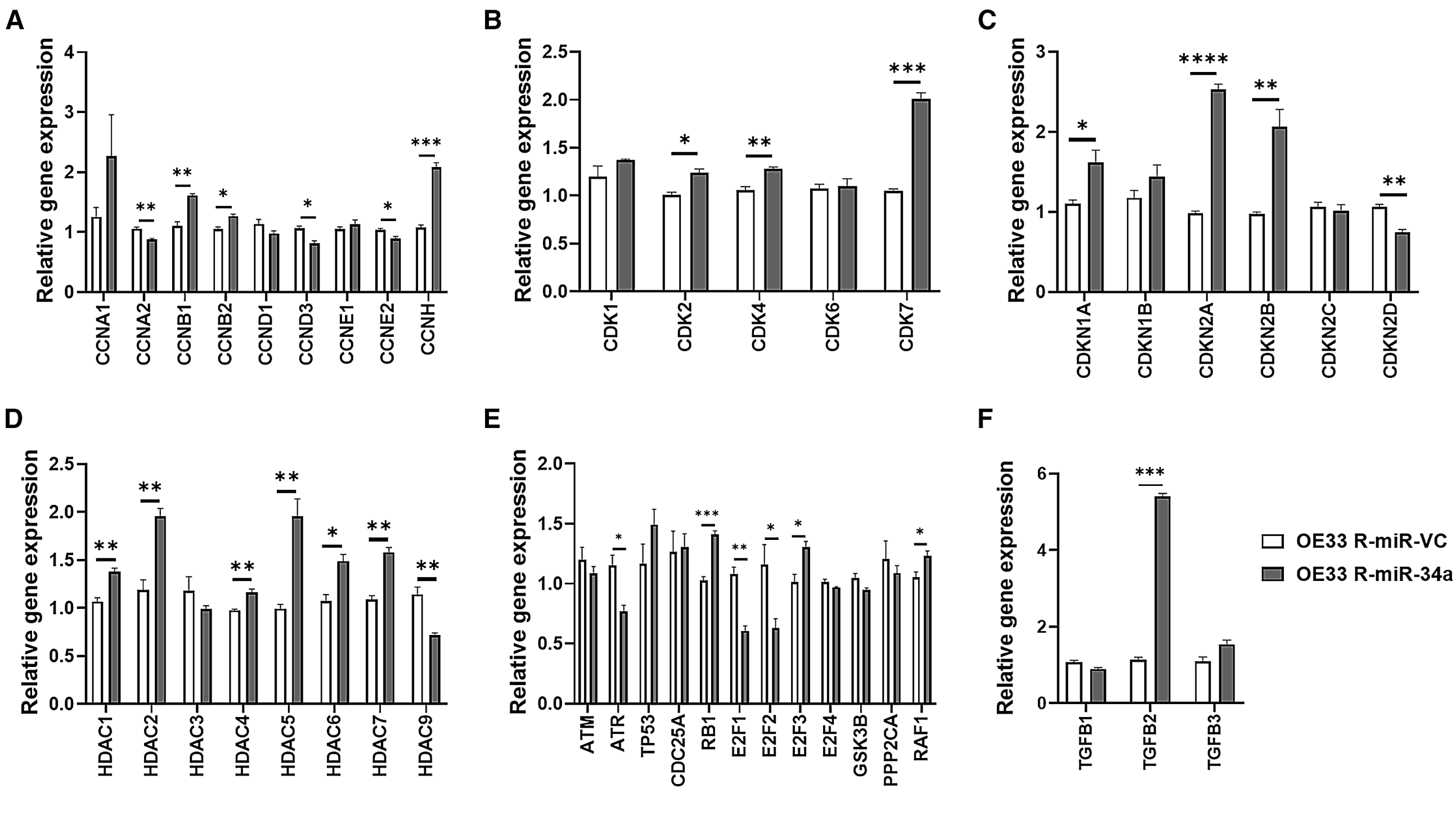

G

	Predicted pairing regions	Seed match	Context++ score	Site type
Position 123-130 of CCNE2 3' UTR	5' ...CCAAUUCACAAGUUACACUGCCA...	8mer	-0.55	Conserved
	|||||||			
**hsa-miR-34a-5p**	3' UGUUGGUCGAUUCUGUGACGGU			

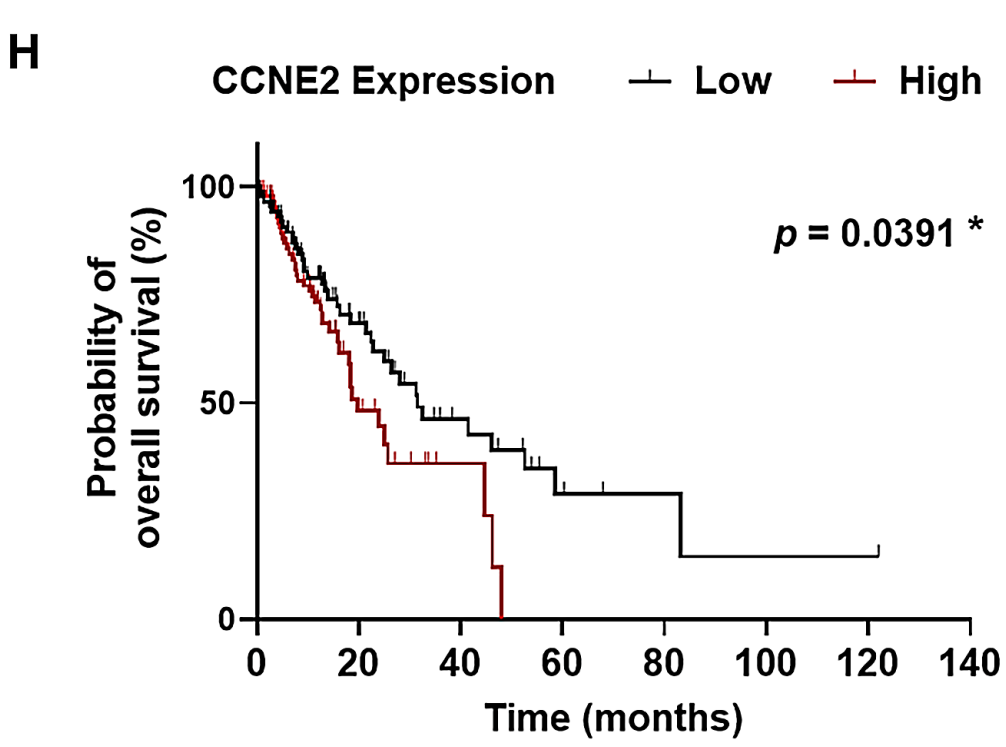

Figure 5. miR-34a overexpression alters expression of 27 cell cycle- and DNA repair-related genes in radioresistant OE33 R EAC cells, and CCNE2 is a predicted target of miR-34a

(A–F) Expression of cell cycle and DNA repair genes was assessed in OE33 R-miR-VC and OE33 R-miR-34a cells by TaqMan PCR arrays; including (A) cyclins, (B) CDKs, (C) CDK inhibitors, (D) HDACs, (E) DNA Damage Response (DDR) and cell cycle-checkpoint regulators, and (F) TGFβ2 growth factors ($n = 3$). Data are presented as mean ± SEM. Statistical analysis was performed by unpaired two-tailed Student's *t* test. $^{*}p < 0.05$, $^{**}p < 0.01$, $^{***}p < 0.001$. *In silico* analysis of miR-34a predicted target genes was performed using TargetScanHuman 8.0. (G) The predicted pairing regions of miR-34a-5p and CCNE2. CCNE2 expression data and survival data from TCGA were collected from FireBrowse and cBioportal, respectively. (H) Kaplan-Meier curves demonstrating the effect of CCNE2 tumoral expression on overall survival outcomes in EAC patients ($n = 181$). CCNE2 levels were separated into two groups; above the median and below the median. Survival is measured in months. Statistical analysis was performed using Kaplan-Meier survival analysis and log rank (Mantel-Cox) test. $^{*}p < 0.05$.

(Figure 5B). Interestingly, the expression of CDK inhibitor genes CDKN1A, CDKN2A, and CDKN2B were significantly increased and expression of CDKN2D was significantly decreased in OE33 R-miR-34a cells, when compared to OE33 R-miR-VC cells (Figure 5C). The expression of histone deacetylases (HDACs) HDAC1, HDAC2, HDAC4, HDAC5, HDAC6, and HDAC7 were significantly increased and expression of HDAC9 was significantly decreased in OE33 R-miR-34a cells, when compared to OE33 R-miR-VC cells (Figure 5D). Among other cell-cycle regulators, expression of ATR, E2F1, and E2F2 were significantly decreased, while RB1, E2F3, RAF1, and TGFβ2 were significantly increased in OE33 R-miR-34a cells, compared to OE33 R-miR-VC cells (Figures 5E and 5F).

Combined, these data demonstrate that miR-34a-5p overexpression results in the altered expression of 27 cell cycle-related genes and further supports alterations in cell cycle as a mechanism underlying miR-34a-mediated radiosensitization of EAC cells.

Downregulation of CCNE2 is a potential mechanism of radiosensitization by miR-34a-5p in EAC

Further investigation into potential mRNA targets of miR-34a-5p was performed by assessing miR-34a-5p predicted target genes using the online prediction tool TargetScanHuman 8.0: https://www.targetscan.org/vert_80/. The CCNE2 gene was demonstrated to be a predictive target of miR-34a-5p, with one highly conserved predicted miR-34a-5p-binding site identified in the CCNE2 sequence (Figure 5G).

The relationship between CCNE2 expression and survival outcomes in EAC patients was investigated using publicly available The Cancer Genome Atlas (TCGA) data. EAC patients with high expression (n = 90) of CCNE2 had significantly worse overall survival (OS) outcomes, when compared to patients with low expression (n = 91) of CCNE2 (p = 0.0391) (median OS of 19.71 vs. 31.54 months) (Figure 5H).

These data support CCNE2 as a potential target of miR-34a-5p in EAC and suggest that loss of miR-34a in EAC may provide a mechanism for treatment resistance and poorer survival via increased expression of CCNE2.

Reduced miR-34a-5p expression in pre-treatment EAC tumor biopsies is associated with advanced pathological T stage, poor pathological response to neo-CRT, and poor prognosis

Having demonstrated that miR-34a-5p expression was decreased *in vitro* and plays a functional role in modulating radioresistance, expression of miR-34a-5p in pre-treatment EAC biopsies was investigated to determine any relationship with clinicopathological parameters. miR-34a-5p expression was assessed in pre-treatment biopsies obtained during diagnostic endoscopy from consenting patients. This analysis was conducted in a pilot cohort (n = 18), followed by validation in an independent cohort (n = 59) of EAC patients. miR-34a-5p expression was assessed by miRNA array in the pilot cohort and by qPCR in the validation cohort. Patient characteristics for the pilot and validation cohorts are presented in Tables 1 and 2, respectively.

miR-34a-5p was expressed in all tumor biopsies assessed. Patients were divided into those achieving a tumor regression grade (TRG) of 1 or 2, which were classified as "good" responders and those achieving a TRG of 3–5, which were classified as "poor" responders, as previously described.[24,28] Supporting our *in vitro* data, miR-34a-5p expression was significantly (p = 0.0155) decreased in pre-treatment tumor biopsies from patients having a subsequent poor pathological response to neo-CRT (n = 10), when compared to patients having a good response (n = 8) (Figure 6A).

These findings were validated in an independent cohort of EAC patients (n = 59), with miR-34a-5p expression in pre-treatment tumor biopsies demonstrated to be significantly (p = 0.0425) decreased in patients who had a poor pathological response to neo-CRT (n = 38), when compared to patients who had a good response (n = 21) (Figure 6B). Patients were also divided into 3 categories based on a 3-point variation of the Mandard TRG,[29] where a TRG of 1 is classified as a complete response (n = 6), TRG 2 and 3 are classified as a partial response (n = 38), and TRG 4 and 5 are classified as no/minimal response (n = 15). miR-34a-5p expression was significantly (p = 0.0062) decreased in patients with no/minimal response, when compared to those having a complete response (Figure 6C). Assessment of miR-34a-5p expression across all TRG scores demonstrated that miR-34a-5p was significantly decreased in pre-treatment tumor biopsies from those who had a TRG 2 (n = 16) (p = 0.0293), TRG 3 (n = 23) (p = 0.002), and TRG 4 (n = 12) (p = 0.0069) when compared to patients who had a pCR (TRG 1) (n = 6) (Figure 6D).

miR-34a-5p expression was not associated with pathological N stage (Figure 6F), clinical T stage (Figure 6G), clinical N stage (Figure 6H), age (Figure 6I), or sex (Figure 6J). However, miR-34a-5p expression was significantly decreased in patients with pathological T stage T1 (p = 0.0127), T2 (p = 0.012), and T3-4 (p = 0.0359), when compared to patients with pathological T stage T0 (Figure 6E). Additionally, patients with decreased expression of miR-34a-5p exhibited a significantly (p = 0.032) reduced probability of overall survival (OS), when compared to those with high miR-34a-5p expression (median OS of 26.79 vs. 72.89 months) (Figure 6K).

To account for potential confounding factors in predicting OS, Cox regression multivariate analysis was performed, including miR-34a-5p expression, TRG, tumor size, tumor differentiation, pathological T stage, and pathological N stage as covariates. Interestingly, only miR-34a-5p expression and pathological T stage were independent predictors of survival after adjusting for these other variables (Table S1). To further refine this analysis, a subsequent Cox regression multivariate analysis was performed, retaining only miR-34a expression and pathological T stage, to assess whether miR-34a-5p expression in combination with pathological T stage remained an independent prognostic factor. Interestingly, both miR-34a-5p

Table 1. Pilot cohort patient characteristics

		Cancer (n = 18)
**Sex**	Male (n)	16
	Female (n)	2
**Age at diagnosis**	median (years)	63
	range (years)	37–75
**Neoadjuvant treatment received**	CRT (n)	18
TRG	1 (n)	3
	2 (n)	5
	3 (n)	0
	4 (n)	8
	5 (n)	2
**Clinical TNM stage**	I	0
	IIa	6
	IIb	2
	III	10
	IV	0

CRT, chemoradiation therapy; TRG, Tumor regression grade; T stage, tumor stage.

Table 2. Validation cohort patient characteristics

		Cancer (n = 18)
**Sex**	Male (n)	49
	Female (n)	8
	Not defined (n)	2
**Age at diagnosis**	median (years)<br>range (years)	63<br>44–79
**Neoadjuvant treatment received**	CRT (n)	59
TRG	1 (n)	6
	2 (n)	15
	3 (n)	23
	4 (n)	12
	5 (n)	3
**Pathological T stage**	0 (n)	5
	1 (n)	10
	2 (n)	9
	3 (n)	31
	4 (n)	1
	Not defined (n)	3
**Clinical T stage**	0 (n)	2
	1 (n)	0
	2 (n)	4
	3 (n)	16
	4 (n)	1
	Not defined (n)	36

CRT, chemoradiation therapy; TRG, Tumor regression grade; T stage, tumor stage.

expression (p = 0.012, hazard ratio [HR] 0.357, 95% CI 0.160–0.796) and pathological T stage (p = 0.035, HR 2.296, 95% CI 1.062–4.962) remained individually predictive of OS.

Although multivariate Cox regression was performed to adjust for the main available clinical parameters, the influence of unmeasured confounders cannot be fully excluded, and the results should therefore be interpreted as an association rather than direct evidence of causality. These data demonstrate that decreased tumoral expression of miR-34a-5p is associated with a poorer pathological response to neo-CRT and a poorer prognosis in EAC and is an independent significant predictor of OS in this cohort. These data suggest that loss of miR-34a may be a mechanism underlying treatment resistance and pathogenesis in EAC patients.

DISCUSSION

The identification of biomarkers predicting response to cytotoxic therapy is crucial for improving the efficacy of cancer treatment and the prognosis of EAC patients. Approximately 50% of all cancer patients receive radiation therapy as part of their treatment regimen and the standard of care treatment for EAC patients often includes concurrent neo-CRT.[30] However, radioresistance remains a significant clinical challenge, with less than 30% of EAC patients achieving a pCR following neo-CRT.[7] For many patients with tumors resistant to neo-CRT, therapeutic options remain limited. Therefore, there is a global unmet need to identify predictive biomarkers of response to neo-CRT and to uncover novel therapeutic targets to enhance treatment response.

Combining chemotherapy with radiation aims to enhance therapeutic outcomes through mechanisms such as spatial cooperation, whereby the systemic effects of chemotherapy enhance the locoregional control of radiation.[31] As radiation resistance is thought to contribute substantially to overall treatment resistance, investigating mechanisms of radioresistance in isolation provides critical insights into the cellular pathways that limit neo-CRT efficacy. In this study, we examined miR-34a-5p using well-established EAC models of radioresistance, including models of acquired radioresistance (OE33 P and OE33 R) and inherent radioresistance (OE33 and OE19), which have previously been used to assess miRNA expression.[22,24] We also utilized a novel cell line model of EAC progression in this study, demonstrating for the first time that FLO-1^{LM} cells, established from FLO-1^{Par} liver metastasis,[26] are significantly more radioresistant than FLO-1^{Par} cells. Thus, this model represents an *in vitro* model of progression and inherent radioresistance in EAC. Although radiation therapy is not included in standard treatment regimens for liver metastases, these findings provide novel insight into the progression of EAC.

Aberrant expression of miRNAs has been associated with resistance to anti-cancer treatment and previous work in our laboratory has demonstrated that miR-31,[24] miR-330-5p,[22] and miR-187[23] are individually associated with resistance to neo-CRT in EAC. Here, we demonstrate that miR-34a-5p expression is significantly decreased

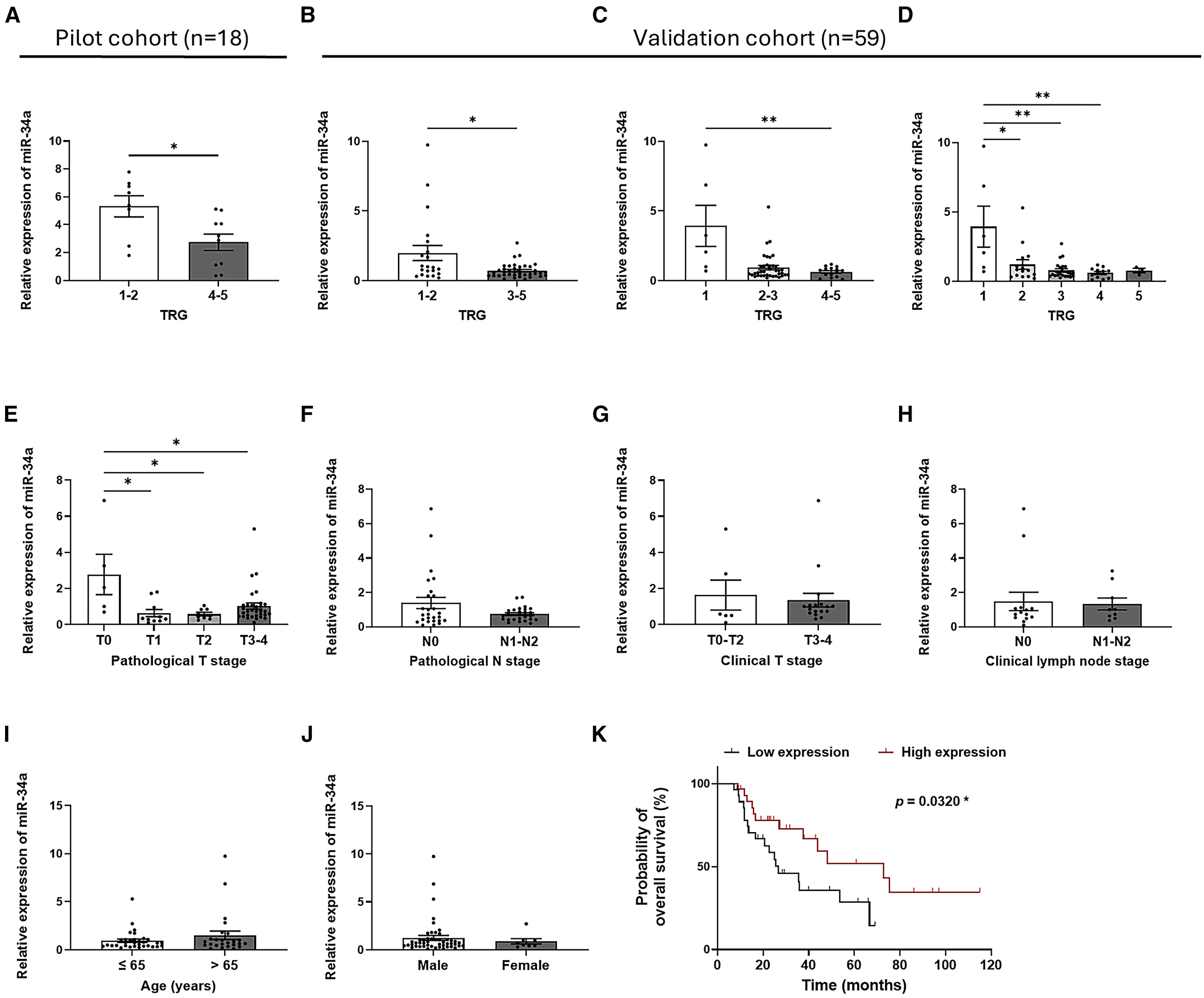

Figure 6. miR-34a-5p expression is decreased in pre-treatment EAC tumor biopsies from patients with poor pathological response to neo-CRT, advanced pathological T stage, and poor OS

Pre-treatment diagnostic tumor biopsies were collected from consenting EAC patients. Patients received neo-CRT. Following surgical resection, the specimen was assigned a TRG by an experienced pathologist. miR-34a expression was assessed in pre-treatment tumor biopsies by qPCR. (A and B) miR-34a-5p expression in (A) a pilot cohort of 18 EAC patients categorized as good responders (TRG 1–2) ($n = 8$) and poor responders (TRG 4–5) ($n = 10$) and (B) an independent cohort of 59 EAC patients categorized as good responders (TRG 1–2) ($n = 21$) and poor responders (TRG 3–5) ($n = 38$). (C and D) Patients were divided into (C) three groups; TRG 1 ($n = 6$), TRG 2–3 ($n = 38$), and TRG 4–5 ($n = 15$) and (D) into 5 groups; TRG 1 ($n = 6$), TRG 2 ($n = 15$), TRG 3 ($n = 24$), TRG 4 ($n = 12$), and TRG 5 ($n = 3$). (E–J) miR-34a-5p expression in (E) patients with pathological T stage T0 ($n = 5$), T1 ($n = 10$), T2 ($n = 9$), and T3-4 ($n = 32$), (F) patients with pathological lymph node stage N0 ($n = 26$), compared to N1-N2 ($n = 26$), (G) patients with clinical T stage T0-2 ($n = 6$), compared to T3-4 ($n = 17$), (H) patients with clinical lymph node stage N0 ($n = 14$), compared to N1-N2 ($n = 9$), (I) patients aged ≤65 y ($n = 32$), compared to >65 y ($n = 25$), and (J) males ($n = 49$), compared to females ($n = 8$). Data are presented as mean ± SEM. Statistical analysis was performed using Mann-Whitney U test. (K) Kaplan-Meier curve demonstrating the effect of miR-34a-5p on OS ($n = 59$) in EAC patients. miR-34a-5p expression levels were separated into two groups; above the median and below the median. Survival is measured in months. Statistical analysis was performed using a Kaplan-Meier survival analysis and log rank (Mantel-Cox) test. *$p < 0.05$, **$p < 0.01$.

in both inherent and acquired radioresistant EAC cell line models, suggesting a consistent role for miR-34a-5p in the radioresponse of EAC. To our knowledge, this is the first study to profile the expression of miR-34a-5p in radioresistant EAC cells. Interestingly, while our findings demonstrate miR-34a-5p downregulation in radioresistance, previous studies in breast cancer and glioblastoma cells have reported associations between increased miR-34a and radioresistance.[32,33] However, these studies have evaluated miR-34a expression following exposure to radiation and indeed, we also demonstrate radiation-induced upregulation of miR-34a-5p in FLO-1[Par]

cells following 2 Gy, indicating a potential involvement of miR-34a in the response to radiation. Supporting these findings, Sasaki et al. demonstrate a dose-dependent increase in miR-34a expression following 30 Gy and 60 Gy.[33] Furthermore, it has been suggested that miR-34a is an indicator of exposure to ionizing radiation.[34] Thus, radiation-induced upregulation of miR-34a-5p likely reflects an acute stress response rather than an underlying radioresistant phenotype, consistent with our findings from unirradiated radioresistant EAC models. Future studies assessing miR-34a-5p expression at later time points following irradiation will be important to determine if these increases are sustained over time in FLO-1^{Par} cells or represent a transient response to radiation exposure.

Supporting our findings *in vitro*, we demonstrated that miR-34a-5p expression is decreased in pre-treatment tumor biopsies from patients who have a subsequent poor response to neo-CRT. This supports a role for miR-34a in the response to cytotoxic therapy and highlights its potential as a novel biomarker of response to treatment in EAC patients. Response to neo-CRT is a predictor of survival in EAC and a pCR is associated with improved OS and DFS.[6] Our data support this, whereby low expression of miR-34a-5p correlates with reduced OS and advanced pathological T stage in these patients. Furthermore, multivariate analysis identified miR-34a-5p as the strongest independent predictor of OS, even surpassing traditional prognostic markers such as TRG. This role for miR-34a is further supported by studies in breast cancer, where loss of miR-34a in is an independent predictor of prognosis in breast cancer tissue[35] and a promising tissue and serum biomarker of poor response to neo-CRT.[36] This detection of miRNAs in the serum offers a less invasive, easily accessible option, and future work is required to assess the potential role of miR-34a as a circulating biomarker predictive of treatment response in EAC. Importantly, while the current study utilized clinically annotated treatment-naive cohorts to investigate miR-34a-5p as a predictive biomarker, future prospective studies incorporating larger multi-center patient cohorts will be essential to further validate the robustness, reproducibility, and clinical applicability of miR-34a-5p as a predictive biomarker of neo-CRT response in EAC.

Given the demonstrated association between decreased miR-34a-5p expression and EAC treatment resistance *in vitro* and *in vivo*, we investigated if miR-34a-5p plays a functional role in modulating the radioresponse in EAC. A stable OE33 R miR-34a-5p overexpression model was generated achieving an approximate 3-fold upregulation, consistent with the increased expression observed in radiosensitive OE33 P cells, compared to OE33 R cells. miR-34a-5p overexpression significantly radiosensitized OE33 R cells to a clinically relevant dose of 2 Gy, demonstrating for the first time that miR-34a-5p functionally modulates radiosensitivity in EAC *in vitro*. While these effects are attributed primarily to miR-34a-5p based on our expression analyses, the precursor-based overexpression system used in this study reflects endogenous miRNA processing and may also permit expression of miR-34a-3p. Supporting our findings, previous studies have identified a functional role for miR-34a in radiosensitizing non-small cell lung cancer (NSCLC) cell lines and murine models.[37,38] Furthermore, in esophageal squamous cell carcinoma (OSCC) cells, miR-34a has been demonstrated to reverse radioresistance by targeting the PI3K/AKT/mTOR pathway.[39]

DNA is the primary target of radiation and thus, DNA damage induction and repair play a crucial role in the response to radiation.[40] While miR-34a-5p did not alter the extent of radiation-induced DNA damage induction, it impaired the ability of OE33 R cells to repair radiation-induced DNA DSBs. Enhanced DNA repair capabilities have long been associated with radioresistance in cancer cells and miR-34a has been demonstrated to directly target DNA repair genes, such as RAD51 in NSCLC and colorectal cancer (CRC) cells, reducing homologous recombination (HR) efficiency and impairing DNA repair.[38,41] Interestingly, both studies also reported miR-34a-mediated DNA damage induction[38,41], however, this was not observed in EAC OE33 R cells in this study. Here, we demonstrate for the first time that overexpression of miR-34a-5p results in decreased expression of ATR in OE33 R cells. ATR is essential for activation of the DDR and its inhibition has previously been demonstrated to increase the radiosensitivity of EAC cells.[42] This downregulation of ATR may therefore represent a mechanism underlying miR-34a-mediated radiosensitization in EAC.

Ionizing radiation induces the activation of cell-cycle checkpoints, which is essential for the repair of DNA damage. Dysregulation of these checkpoints can lead to ineffective repair of radiation-induced damage, ultimately enhancing the effectiveness of radiation therapy.[43] miR-34a is a known regulator of cell cycle, inducing cell-cycle arrest through the p53 signaling pathway.[20,44,45] Here, we demonstrate that miR-34a-5p impaired the progression of OE33 R cells through the G0/G1 phase following irradiation, while simultaneously limiting radiation-induced G2/M phase arrest. G0/G1 arrest limits the amount of cells progressing through the cell cycle, leading to diminished cell division and overall growth. Importantly, the G2/M checkpoint is a critical control mechanism for repairing DNA damage before entry into mitosis.[46] Therefore, the attenuation of the G2/M arrest following miR-34a-5p overexpression may contribute to the impaired repair of radiation-induced DNA damage, making cells more susceptible to radiation-induced death. Cells in the G1 and G2/M phases are typically more radiosensitive,[47] thus, the effect of miR-34a-5p on these checkpoints likely contributes to enhanced radiosensitivity in OE33 R cells. Specifically, we demonstrated that miR-34a-5p overexpression resulted in increased expression of cell-cycle regulators, such as cyclin B1, cyclin B2, and CDK2, which facilitate progression through the G2/M phase.[46] However, genes which were decreased following miR-34a-5p overexpression were of particular interest as miRNAs typically function by downregulating target mRNAs through degradation or repression of translation.[8] Among these, CCNE2 emerged as particularly interesting as, not only did miR-34a-5p overexpression decrease expression of CCNE2, but CCNE2 also contains a highly conserved potential miR-34a-5p-binding site, supporting the idea that it may be a direct

target of miR-34a-5p. CCNE2 encodes cyclin E2, a key regulator of the G1/S transition, and its dysregulation has been implicated in breast cancer, where it contributes to uncontrolled proliferation, genomic instability, and treatment resistance.[48,49] Furthermore, analysis of TCGA demonstrated that low expression of CCNE2 in EAC tumors was associated with poor OS outcomes, further highlighting its potential role as an independent factor influencing patient outcomes. These findings support a model whereby CCNE2 is a direct target of miR-34a-5p, which may provide a mechanism for miR-34a-mediated radiosensitization of OE33 R EAC cells, providing a mechanistic link between miR-34a activity, cell cycle regulation, and therapeutic response. While these findings support a functional role for miR-34a-5p in modulating radiosensitivity in EAC, future strand-specific studies (e.g., miRNA mimics) will be important to further investigate the individual contributions of miR-34a-5p and miR-34a-3p to these phenotypic effects. In addition, although our findings support a functional association between miR-34a-5p and CCNE2 expression, further mechanistic studies, such as luciferase reporter or Ago2-RIP/CLIP approaches may provide additional insight into potential binding and direct regulation of CCNE2 by miR-34a-5p. Furthermore, the current study focused primarily on intrinsic cellular determinants of radiosensitivity, including DNA damage repair and cell-cycle regulation. Therefore, future investigations using more complex *in vitro* and *in vivo* models will be important to determine whether miR-34a-5p also influences the tumor microenvironment, immune modulation, and additional mechanisms contributing to therapeutic response in EAC.

Our findings suggest that miR-34a may serve as a valuable target for therapeutic intervention aimed at reversing radioresistance and improving treatment outcomes in EAC patients. Interestingly, miR-34a replacement has previously been investigated as a cancer therapy in a phase 1 clinical trial (NCT01829971) for the treatment of solid tumors due to its role in reducing cell proliferation, migration and invasion.[50] Additionally, it demonstrated synergistic effects with anti-cancer therapies, further supporting our data. While the current study demonstrates that restoration of miR-34a-5p enhances radiosensitivity *in vitro*, further preclinical and translational studies are required for clinical implementation, including optimizsation of miR-34a delivery approaches, such as nanoparticle-, liposomal-, or viral-based systems, to achieve effective tumor-specific delivery. Given the defined schedule of neo-CRT in EAC patients, miR-34a-based therapies would likely require administration prior to or concurrently with radiation therapy to maximize radiosensitization. However, the optimal route, timing, and dosing strategy remain to be determined. Importantly, the current findings are limited to *in vitro* models and therefore require further validation *in vivo* using xenograft and patient-derived models to assess therapeutic efficacy, safety, and tolerability. Prospective clinical studies will ultimately be necessary to determine whether miR-34a-based strategies can improve treatment response and clinical outcomes in EAC patients receiving neo-CRT.

This study highlights miR-34a as a novel tumoral biomarker predicting response to neo-CRT in EAC for improved patient stratification. Additionally, our data highlight miR-34a as a radiosensitiser in EAC, suggesting a novel therapeutic strategy to enhance the efficacy of neo-CRT in EAC patients.

MATERIALS AND METHODS

Cell lines and cell culture

OE33 and OE19 EAC cell lines were obtained from the European Collection of Cell Culture (ECACC, UK). The isogenic EAC radioresistance model (OE33 R and OE33 P) was generated in our laboratory as previously described.[25] OE19, OE33, OE33 P, and OE33 R cells were cultured in RPMI-1640 medium (Gibco) supplemented with 10% (v/v) fetal bovine serum (FBS) (Gibco). FLO-1^{Par} and FLO-1^{LM} cell lines were gifted from Dr. Nicholas Clemons (Tumorigenesis & Cancer Therapeutics Lab, Peter MacCallum Cancer Center, Victoria, Australia).[26] FLO-1^{Par} and FLO-1^{LM} cell lines were cultured in DMEM medium with 4.5 g/L glucose and L-glutamine, supplemented with 10% (v/v) FBS. All cell lines were maintained at 37°C, 5% CO_2/95% humidified air.

Irradiation

X-ray irradiation at a dose rate of 1.74 Gy/min was performed using an Xstrahl RS225 X-ray irradiator (Xstrahl, Walsall, United Kingdom). Cells were placed in the center of the X-ray irradiator and were irradiated uniformly at the center of the beam at a distance of 40 cm with a beam produced by 200 kV and 15 mA. Control plates were mock irradiated by placing at room temperature (RT) for the duration.

Clonogenic assay

EAC cells were seeded in 6-well plates in complete media at optimized seeding densities for the ionizing radiation dose (Table S2) and incubated at 37°C, 5% CO_2/95% humidified air. At 24 h following seeding, cells were irradiated or mock irradiated and further incubated at 37°C, 5% CO_2/95% humidified air for 7–10 days until colonies had formed but not merged. Colonies were fixed and stained using 0.1% w/v crystal violet, 60% v/v methanol (MeOH) (Honeywell, South Carolina, United States). Cells were gently washed with H_2O and left overnight to air dry. Colonies were counted using a GelCount colony counter (Oxford Optronix Ltd, Abingdon, United Kingdom). Optimized CHARM (compact Hough and radial map) image processing algorithms were used for each cell line to detect colonies consisting of at least 50 cells. Plating efficiency (PE) was calculated using the formula: PE = no. of colonies/no. of cells seeded. The surviving fraction was calculated using the formula: Surviving fraction = no. of colonies/(no. of cells seeded × PE).[51]

RNA isolation

RNA was isolated from EAC cell lines using TRIzol Reagent (Thermo Fisher Scientific, Massachusetts, United States) according to the manufacturer's instructions. RNA was isolated from EAC tissue biopsies using the miRNeasy Mini Kit (QIAGEN, Hilden, Germany) according to the manufacturer's instructions. RNA was quantified spectrophotometrically using a DS-11 Series Spectrophotometer/Fluorometer (DeNovix, Delaware, United States).

qPCR

Complementary DNA (cDNA) synthesis from RNA was performed by reverse transcription using the miRCURY LNA RT Kit (QIAGEN) as per the manufacturer's guidelines. qPCR for quantification of miR-34a-5p expression was carried out using the miRCURY LNA SYBR Green PCR Kit (QIAGEN) and hsa-miR-34a-5p miRCURY LNA primers. qPCR was performed using a Quant Studio 5 Real-Time PCR System (Applied Biosystems). U6 was used as an endogenous control for data normalization. Data analysis was performed on the Thermo Fisher Connect Platform using the Livak method, $2^{-\Delta\Delta Ct}$, using one sample as the calibrator for analysis.[52]

Stable miR-34a overexpression

Stable miR-34a overexpression was performed using overexpression plasmid (MI0000268) (Origene, Herford, Germany) or vector control (VC) plasmid (pCMVMIR). Cells were seeded at a density of 3×10^5 cells in a 100 mm tissue culture dish, and incubated at 37°C, 5% CO_2/95% humidified air overnight. Cells were transfected with plasmids (5 μg) using Lipofectamine 2000 transfection reagent (6 μL) (Invitrogen) and Opti-MEM medium (Gibco), and incubated at 37°C, 5% CO_2/95% humidified air for 4–6 h, at which time Opti-MEM was replaced with fresh complete medium. At 24 h following transfection, selective antibiotic (geneticin) was added. Cells were maintained under a 450 μg/mL G418 (geneticin) (Thermo Fisher Scientific) selection for 21 days.

Cell cycle and DNA damage assessment by flow cytometry

Cells were stained for DNA double-strand breaks using FITC anti-H_2A.X Phospho (Ser139) Antibody (BioLegend) (1:100 in PBS/FBS [2%] [v/v]/0.1% [v/v] Triton X-100) and incubated in the dark, for 2 h at RT. Cells irradiated with 10 Gy X-ray radiation were used as a positive control. Cells were washed twice with PBS/FBS (2%), resuspended in PI staining solution (PI [0.025 mg/mL]/RNAse A [0.1 mg/mL]/Triton X-100 [0.1%)/PBS]), and incubated in the dark for 30 min at 37°C to assess cell cycle. Samples were acquired using a BD FACSCanto II (Becton Dickinson and Company) and FACSDiva Software (Becton Dickinson and Company). Analysis was performed using FlowJo v.10 Software (Becton Dickinson and Company).

Gene arrays

cDNA was synthesized from RNA using the BioScript RT reaction (Meridian Bioscience, Ohio, United States) according to manufacturer's guidelines. The expression profile of a panel of cell cycle-related genes was assessed using TaqMan Gene Expression Arrays (Applied Biosystems) and TaqMan Universal PCR Master Mix (Thermo Fisher Scientific). The averaged expression of 18S, GAPDH, GUSB, and HPRT1 endogenous control genes was used for data normalization. qPCR was performed using a Quant Studio 5 Real-Time PCR System (Applied Biosystems). Data analysis was performed on the Thermo Fisher Connect Platform. Relative changes in mRNA expression were assessed using the Livak method.[52]

In silico miRNA target prediction

TargetScanHuman 8.0 was used to identify predicted miR-34a-5p target genes. hsa-miR-34a-5p was queried in TargetScanHuman, which identifies predicted target genes based on conserved and non-conserved seed sequence complementarity within 3′ UTRs. The output includes predicted binding sites classified according to seed match type (e.g., 7-mer or 8-mer sites) and associated context++ scores, which reflect predicted binding efficacy.

Publicly available patient data collection and survival assessment

Patients were selected from TCGA consortium (PanCancer Atlas).[53,54] cBioPortal: www.cbioportal.org[55] was used to download survival and gene expression TCGA data for 181 EAC patients. RNA sequencing (RNA-seq) count data were obtained from the Firebrowse portal.[56] Survival analysis was performed using Kaplan-Meier curves to evaluate the time to event. Patients that did not have an event occurrence were censored. The median expression values were used to stratify patients into high expression (values > median) and low expression (values < median). Log-rank (Mantel-Cox) analysis was performed to assess the relationship between expression and OS.

Patient recruitment, ethical approval, and sample collection

Following ethical approval from the joint St James's Hospital/AMNCH ethical review board (REC reference number: 041113/10804) and written informed consent, pre-treatment tumor tissue biopsies were obtained from EAC patients attending St James's Hospital, Dublin between June 2004 and August 2017). EAC pre-treatment biopsies were obtained during endoscopy prior to neo-CRT. Immediately adjacent tissue was assessed by an experienced pathologist and histologically confirmed to be tumor by hematoxylin and eosin staining.

Patient treatment

EAC patients recruited in this study were treated based on the prevailing national guidelines, according to the National Cancer Control Program (NCCP) National Systemic Anti-Cancer Therapy (SACT) Regimens, with CROSS neo-CRT regimen, followed by radical surgery. Treatment involved five cycles of carboplatin and paclitaxel administered alongside RT, delivered in 23 fractions of 1.8 Gy (total radiation dose of 41.4 Gy). Tumor response to neo-CRT was assessed pathologically by an experienced pathologist who assigned a TRG using the Mandard Grading System.[57]

miRNA arrays

miRNA expression in pilot cohort tumor samples was performed using miRNA arrays. miRNA (40 ng) was reverse transcribed into cDNA using Universal RT primers and the miRNA Reverse Transcription Kit (Exiqon) as per the manufacturer's instructions. Global miRNA expression profiling was performed using the human miRCURY LNA Universal RT miRNA PCR panels I and II (Exiqon). miRNA arrays were performed using the 7900HT Real-Time PCR System (Applied Biosystems). Analysis was performed using

GenEx 5.0 software (MultiD Analyses AB). Data were normalized using the global mean and one sample was set as the calibrator for relative quantification analysis.

Statistical analysis

GraphPad Prism 10 software (GraphPad, California, United States) was used to perform statistical analysis. Experiments were repeated at least three times for statistical assessment and results were displayed as mean ± SEM, unless otherwise indicated. For comparisons between multiple experimental groups significance was determined by analysis of variance (ANOVA) with post-hoc Tukey's multiple comparisons testing. For comparisons between two groups, significance was determined by two-tailed Student's *t* test. Both unpaired *t* tests (for independent samples) and paired *t* tests (for matched or dependent samples) were applied where appropriate, as indicated in the figure legends. γH_2AX intensity was analyzed using a linear mixed-effects model with fixed effects for cell line (OE33 R-miR-VC vs. OE33 R-miR-34a), dose (0 Gy vs. 2 Gy), time (20 min, 6 h, 10 h, 24 h; categorical) and all interactions, and a random intercept for biological replicate (replicate_id). Models were fitted using the "lme4" (v.2.0-1) and "lmerTest" (v.3.2-1) packages in RStudio (v.2026.04.0). Type-III F-tests with Satterthwaite (or Kenward-Roger) degrees of freedom were used to assess fixed effects. Post-hoc pairwise contrasts between cell lines at each dose-time combination were obtained from model-based estimated marginal means (emmeans), with 95% confidence intervals and *p* values adjusted for multiple testing using the Benjamini-Hochberg method. Analysis on patient samples utilized Mann-Whitney U test. Kaplan-Meier survival analysis was used to assess survival. Multivariate analysis was performed using IBM SPSS Statistics (version 24). Results were considered significant where probability (p) ≤ 0.05.

DATA AND CODE AVAILABILITY

The data supporting this study are available upon request from the corresponding author.

ACKNOWLEDGMENTS

The authors would like to thank the patients and their families for their participation in this study. We also thank Dr. Nicholas Clemons (Tumorigenesis & Cancer Therapeutics Lab, Peter MacCallum Cancer Center, Victoria, Australia) for gifting FLO-1^{Par} and FLO-1^{LM} cells for use in this study. We thank Dr. Abdullah Alhusaini for his contribution to data analysis. C. Cahill was supported by a Trinity College Dublin Provost PhD Scholarship. N. Lynam-Lennon was supported by the Health Research Board (Ireland), grant number: EIA-2017-020.

AUTHOR CONTRIBUTIONS

C.C., conceptualization, data curation, formal analysis, investigation, methodology, resources, software, validation, visualization, writing – original draft, and writing – review and editing; D.H., investigation, methodology, resources, and writing – review and editing; L.E.K., formal analysis and writing – review and editing; J.J.P., formal analysis; J.M., investigation, methodology, and resources; M.Ó.M., formal analysis, visualization, and writing – review and editing; A.C., data curation, investigation, and resources; A.B.H., data curation, resources, and writing – review and editing; A.K., investigation and methodology; R.L., investigation and methodology; R.M.O., investigation and methodology; W.-L.W.W., data curation and investigation; M.S.M., data curation and project administration; O.M.D., resources; A.T., resources; N.R., resources; C.D., resources; D.O., resources; C.M., resources; A.M., resources; J.L., conceptualization, formal analysis, and writing – review and editing; M.A.L., resources; J.V.R., resources; S.G.M., conceptualization, formal analysis, and validation; J.O., conceptualization, project administration, and supervision; N.L.-L., conceptualization, formal analysis, funding acquisition, project administration, supervision, validation, and writing – review and editing.

DECLARATION OF INTERESTS

The authors declare no competing interests.

SUPPLEMENTAL INFORMATION

Supplemental information can be found online at https://doi.org/10.1016/j.omtn.2026.103064.

REFERENCES

1. Sung, H., Ferlay, J., Siegel, R.L., Laversanne, M., Soerjomataram, I., Jemal, A., and Bray, F. (2021). Global Cancer Statistics 2020: GLOBOCAN Estimates of Incidence and Mortality Worldwide for 36 Cancers in 185 Countries. CA Cancer J. Clin. *71*, 209–249. https://doi.org/10.3322/caac.21660.

2. O'Sullivan, J., Lysaght, J., Donohoe, C.L., and Reynolds, J.V. (2018). Obesity and gastrointestinal cancer: the interrelationship of adipose and tumour microenvironments. Nat. Rev. Gastroenterol. Hepatol. *15*, 699–714. https://doi.org/10.1038/s41575-018-0069-7.

3. Allemani, C., Matsuda, T., Di Carlo, V., Harewood, R., Matz, M., Nikšić, M., Bonaventure, A., Valkov, M., Johnson, C.J., Estève, J., et al. (2018). Global surveillance of trends in cancer survival 2000-14 (CONCORD-3): analysis of individual records for 37 513 025 patients diagnosed with one of 18 cancers from 322 population-based registries in 71 countries. Lancet *391*, 1023–1075. https://doi.org/10.1016/S0140-6736(17)33326-3.

4. Codipilly, D.C., Sawas, T., Dhaliwal, L., Johnson, M.L., Lansing, R., Wang, K.K., Leggett, C.L., Katzka, D.A., and Iyer, P.G. (2021). Epidemiology and Outcomes of Young-Onset Esophageal Adenocarcinoma: An Analysis from a Population-Based Database. Cancer Epidemiol. Biomarkers Prev. *30*, 142–149. https://doi.org/10.1158/1055-9965.EPI-20-0944.

5. Shapiro, J., van Lanschot, J.J.B., Hulshof, M.C.C.M., van Hagen, P., van Berge Henegouwen, M.I., Wijnhoven, B.P.L., van Laarhoven, H.W.M., Nieuwenhuijzen, G.A.P., Hospers, G.A.P., Bonenkamp, J.J., et al. (2015). Neoadjuvant chemoradiotherapy plus surgery versus surgery alone for oesophageal or junctional cancer (CROSS): long-term results of a randomised controlled trial. Lancet Oncol. *16*, 1090–1098. https://doi.org/10.1016/S1470-2045(15)00040-6.

6. Reynolds, J.V., Muldoon, C., Hollywood, D., Ravi, N., Rowley, S., O'Byrne, K., Kennedy, J., and Murphy, T.J. (2007). Long-term outcomes following neoadjuvant chemoradiotherapy for esophageal cancer. Ann. Surg. *245*, 707–716. https://doi.org/10.1097/01.sla.0000254367.15810.38.

7. Herskovic, A., Russell, W., Liptay, M., Fidler, M.J., and Al-Sarraf, M. (2012). Esophageal carcinoma advances in treatment results for locally advanced disease: review. Ann. Oncol. *23*, 1095–1103. https://doi.org/10.1093/annonc/mdr433.

8. Hutvágner, G., and Zamore, P.D. (2002). A microRNA in a multiple-turnover RNAi enzyme complex. Science *297*, 2056–2060. https://doi.org/10.1126/science.1073827.

9. Lewis, B.P., Shih, I.H., Jones-Rhoades, M.W., Bartel, D.P., and Burge, C.B. (2003). Prediction of mammalian microRNA targets. Cell *115*, 787–798. https://doi.org/10.1016/s0092-8674(03)01018-3.

10. Bommer, G.T., Gerin, I., Feng, Y., Kaczorowski, A.J., Kuick, R., Love, R.E., Zhai, Y., Giordano, T.J., Qin, Z.S., Moore, B.B., et al. (2007). p53-mediated activation of miRNA34 candidate tumor-suppressor genes. Curr. Biol. *17*, 1298–1307. https://doi.org/10.1016/j.cub.2007.06.068.

11. Raver-Shapira, N., Marciano, E., Meiri, E., Spector, Y., Rosenfeld, N., Moskovits, N., Bentwich, Z., and Oren, M. (2007). Transcriptional activation of miR-34a contributes to p53-mediated apoptosis. Mol. Cell *26*, 731–743. https://doi.org/10.1016/j.molcel.2007.05.017.

12. Craig, V.J., Cogliatti, S.B., Imig, J., Renner, C., Neuenschwander, S., Rehrauer, H., Schlapbach, R., Dirnhofer, S., Tzankov, A., and Müller, A. (2011). Myc-mediated repression of microRNA-34a promotes high-grade transformation of B-cell

lymphoma by dysregulation of FoxP1. Blood *117*, 6227–6236. https://doi.org/10.1182/blood-2010-10-312231.

13. Xiong, S., Hu, M., Li, C., Zhou, X., and Chen, H. (2019). Role of miR-34 in gastric cancer: From bench to bedside. Oncol. Rep. *42*, 1635–1646. https://doi.org/10.3892/or.2019.7280.

14. Chen, P.C., Yu, C.C., Huang, W.Y., Huang, W.H., Chuang, Y.M., Lin, R.I., Lin, J.M.J., Lin, H.Y., Jou, Y.C., Shen, C.H., and Chan, M.W.Y. (2019). c-Myc Acts as a Competing Endogenous RNA to Sponge miR-34a, in the Upregulation of CD44, in Urothelial Carcinoma. Cancers (Basel) *11*, 1457. https://doi.org/10.3390/cancers11101457.

15. Cao, W., Yang, W., Fan, R., Li, H., Jiang, J., Geng, M., Jin, Y., and Wu, Y. (2014). miR-34a regulates cisplatin-induce gastric cancer cell death by modulating PI3K/AKT/survivin pathway. Tumour Biol. *35*, 1287–1295. https://doi.org/10.1007/s13277-013-1171-7.

16. Liu, C., Zhou, C., Gao, F., Cai, S., Zhang, C., Zhao, L., Zhao, F., Cao, F., Lin, J., Yang, Y., et al. (2011). MiR-34a in age and tissue related radio-sensitivity and serum miR-34a as a novel indicator of radiation injury. Int. J. Biol. Sci. *7*, 221–233. https://doi.org/10.7150/ijbs.7.221.

17. Li, L., Story, M., and Legerski, R.J. (2001). Cellular responses to ionizing radiation damage. Int. J. Radiat. Oncol. Biol. Phys. *49*, 1157–1162. https://doi.org/10.1016/s0360-3016(00)01524-8.

18. Liu, Y.P., Zheng, C.C., Huang, Y.N., He, M.L., Xu, W.W., and Li, B. (2020). Molecular mechanisms of chemo- and radiotherapy resistance and the potential implications for cancer treatment. MedComm *2*, 315–340. https://doi.org/10.1002/mco2.55.

19. Kofman, A.V., Kim, J., Park, S.Y., Dupart, E., Letson, C., Bao, Y., Ding, K., Chen, Q., Schiff, D., Larner, J., and Abounader, R. (2013). microRNA-34a promotes DNA damage and mitotic catastrophe. Cell Cycle *12*, 3500–3511. https://doi.org/10.4161/cc.26459.

20. Sun, F., Fu, H., Liu, Q., Tie, Y., Zhu, J., Xing, R., Sun, Z., and Zheng, X. (2008). Downregulation of CCND1 and CDK6 by miR-34a induces cell cycle arrest. FEBS Lett. *582*, 1564–1568. https://doi.org/10.1016/j.febslet.2008.03.057.

21. Hermeking, H. (2010). The miR-34 family in cancer and apoptosis. Cell Death Differ. *17*, 193–199. https://doi.org/10.1038/cdd.2009.56.

22. Bibby, B.A.S., Reynolds, J.V., and Maher, S.G. (2015). MicroRNA-330-5p as a Putative Modulator of Neoadjuvant Chemoradiotherapy Sensitivity in Oesophageal Adenocarcinoma. PLoS One *10*, e0134180. https://doi.org/10.1371/journal.pone.0134180.

23. Lynam-Lennon, N., Bibby, B.A., Mongan, A.M., Marignol, L., Paxton, C.N., Geiersbach, K., Bronner, M.P., O'Sullivan, J., Reynolds, J., and Maher, S.G. (2016). Low miR-187 expression promotes resistance to chemoradiation therapy in vitro and correlates with treatment failure in patients with esophageal adenocarcinoma. Mol. Med. *22*, 388–397. https://doi.org/10.2119/molmed.2016.00020.

24. Lynam-Lennon, N., Reynolds, J.V., Marignol, L., Sheils, O.M., Pidgeon, G.P., and Maher, S.G. (2012). MicroRNA-31 modulates tumour sensitivity to radiation in oesophageal adenocarcinoma. J. Mol. Med. *90*, 1449–1458. https://doi.org/10.1007/s00109-012-0924-x.

25. Lynam-Lennon, N., Reynolds, J.V., Pidgeon, G.P., Lysaght, J., Marignol, L., and Maher, S.G. (2010). Alterations in DNA repair efficiency are involved in the radioresistance of esophageal adenocarcinoma. Radiat. Res. *174*, 703–711. https://doi.org/10.1667/RR2295.1.

26. Liu, D.S., Hoefnagel, S.J.M., Fisher, O.M., Krishnadath, K.K., Montgomery, K.G., Busuttil, R.A., Colebatch, A.J., Read, M., Duong, C.P., Phillips, W.A., and Clemons, N.J. (2016). Novel metastatic models of esophageal adenocarcinoma derived from FLO-1 cells highlight the importance of E-cadherin in cancer metastasis. Oncotarget *7*, 83342–83358. https://doi.org/10.18632/oncotarget.13391.

27. Mah, L.J., El-Osta, A., and Karagiannis, T.C. (2010). gammaH2AX: a sensitive molecular marker of DNA damage and repair. Leukemia *24*, 679–686. https://doi.org/10.1038/leu.2010.6.

28. Lynam-Lennon, N., Maher, S.G., Maguire, A., Phelan, J., Muldoon, C., Reynolds, J.V., and O'Sullivan, J. (2014). Altered mitochondrial function and energy metabolism is associated with a radioresistant phenotype in oesophageal adenocarcinoma. PLoS One *9*, e100738. https://doi.org/10.1371/journal.pone.0100738.

29. Donohoe, C.L., O'Farrell, N.J., Grant, T., King, S., Clarke, L., Muldoon, C., and Reynolds, J.V. (2013). Classification of pathologic response to neoadjuvant therapy in esophageal and junctional cancer: assessment of existing measures and proposal of a novel 3-point standard. Ann. Surg. *258*, 784–792. https://doi.org/10.1097/SLA.0b013e3182a66588.

30. Baskar, R., Lee, K.A., Yeo, R., and Yeoh, K.W. (2012). Cancer and radiation therapy: current advances and future directions. Int. J. Med. Sci. *9*, 193–199. https://doi.org/10.7150/ijms.3635.

31. Steel, G.G., and Peckham, M.J. (1979). Exploitable mechanisms in combined radiotherapy-chemotherapy: the concept of additivity. Int. J. Radiat. Oncol. Biol. Phys. *5*, 85–91.

32. Kato, M., Paranjape, T., Müller, R.U., Nallur, S., Gillespie, E., Keane, K., Esquela-Kerscher, A., Weidhaas, J.B., and Slack, F.J. (2009). The mir-34 microRNA is required for the DNA damage response in vivo in C. elegans and in vitro in human breast cancer cells. Oncogene *28*, 2419–2424. https://doi.org/10.1038/onc.2009.106.

33. Sasaki, A., Udaka, Y., Tsunoda, Y., Yamamoto, G., Tsuji, M., Oyamada, H., Oguchi, K., and Mizutani, T. (2012). Analysis of p53 and miRNA expression after irradiation of glioblastoma cell lines. Anticancer Res. *32*, 4709–4713.

34. Halimi, M., Shahabi, A., Moslemi, D., Parsian, H., Asghari, S.M., Sariri, R., Yeganeh, F., and Zabihi, E. (2016). Human serum miR-34a as an indicator of exposure to ionizing radiation. Radiat. Environ. Biophys. *55*, 423–429. https://doi.org/10.1007/s00411-016-0661-6.

35. Agarwal, S., Hanna, J., Sherman, M.E., Figueroa, J., and Rimm, D.L. (2015). Quantitative assessment of miR34a as an independent prognostic marker in breast cancer. Br. J. Cancer *112*, 61–68. https://doi.org/10.1038/bjc.2014.573.

36. Shaban, N.Z., Ibrahim, N.K., Saada, H.N., El-Rashidy, F.H., Shaaban, H.M., Farrag, M.A., ElDebaiky, K., and Kodous, A.S. (2022). miR-34a and miR-21 as biomarkers in evaluating the response of chemo-radiotherapy in Egyptian breast cancer patients. J. Radiat. Res. Appl. Sci. *15*, 285–292. https://doi.org/10.1016/j.jrras.2022.08.001.

37. Duan, W., Xu, Y., Dong, Y., Cao, L., Tong, J., and Zhou, X. (2013). Ectopic expression of miR-34a enhances radiosensitivity of non-small cell lung cancer cells, partly by suppressing the LyGDI signaling pathway. J. Radiat. Res. *54*, 611–619. https://doi.org/10.1093/jrr/rrs136.

38. Cortez, M.A., Valdecanas, D., Niknam, S., Peltier, H.J., Diao, L., Giri, U., Komaki, R., Calin, G.A., Gomez, D.R., Chang, J.Y., et al. (2015). In Vivo Delivery of miR-34a Sensitizes Lung Tumors to Radiation Through RAD51 Regulation. Mol. Ther. Nucleic Acids *4*, e270. https://doi.org/10.1038/mtna.2015.47.

39. Ye, Z., Xie, T., Yan, F., Wang, L., Fang, J., Wang, Z., Hu, F., Wang, F., and Fu, Z. (2021). MiR-34a reverses radiation resistance on ECA-109 cells by inhibiting PI3K/AKT/mTOR signal pathway through downregulating the expression of SIRT1. Int. J. Radiat. Biol. *97*, 452–463. https://doi.org/10.1080/09553002.2021.1866225.

40. Jackson, S.P., and Bartek, J. (2009). The DNA-damage response in human biology and disease. Nature *461*, 1071–1078. https://doi.org/10.1038/nature08467.

41. Chen, S., Liu, R., Wang, Q., Qi, Z., Hu, Y., Zhou, P., and Wang, Z. (2019). MiR-34s negatively regulate homologous recombination through targeting RAD51. Arch. Biochem. Biophys. *666*, 73–82. https://doi.org/10.1016/j.abb.2019.03.017.

42. Leszczynska, K.B., Dobrynin, G., Leslie, R.E., Ient, J., Boumelha, A.J., Senra, J.M., Hawkins, M.A., Maughan, T., Mukherjee, S., and Hammond, E.M. (2016). Preclinical testing of an Atr inhibitor demonstrates improved response to standard therapies for esophageal cancer. Radiother. Oncol. *121*, 232–238. https://doi.org/10.1016/j.radonc.2016.10.023.

43. Pawlik, T.M., and Keyomarsi, K. (2004). Role of cell cycle in mediating sensitivity to radiotherapy. Int. J. Radiat. Oncol. Biol. Phys. *59*, 928–942. https://doi.org/10.1016/j.ijrobp.2004.03.005.

44. Ji, Q., Hao, X., Meng, Y., Zhang, M., Desano, J., Fan, D., and Xu, L. (2008). Restoration of tumor suppressor miR-34 inhibits human p53-mutant gastric cancer tumorspheres. BMC Cancer *8*, 266. https://doi.org/10.1186/1471-2407-8-266.

45. Guessous, F., Zhang, Y., Kofman, A., Catania, A., Li, Y., Schiff, D., Purow, B., and Abounader, R. (2010). microRNA-34a is tumor suppressive in brain tumors and glioma stem cells. Cell Cycle *9*, 1031–1036. https://doi.org/10.4161/cc.9.6.10987.

46. Stark, G.R., and Taylor, W.R. (2004). Analyzing the G2/M checkpoint. Methods Mol. Biol. *280*, 51–82.

47. Sinclair, W.K., and Morton, R.A. (1966). X-ray sensitivity during the cell generation cycle of cultured Chinese hamster cells. Radiat. Res. *29*, 450–474.

48. Lee, C., Fernandez, K.J., Alexandrou, S., Sergio, C.M., Deng, N., Rogers, S., Burgess, A., and Caldon, C.E. (2020). Cyclin E2 Promotes Whole Genome Doubling in Breast Cancer. Cancers (Basel) *12*, 2268. https://doi.org/10.3390/cancers12082268.

49. Caldon, C.E., Sergio, C.M., Kang, J., Muthukaruppan, A., Boersma, M.N., Stone, A., Barraclough, J., Lee, C.S., Black, M.A., Miller, L.D., et al. (2012). Cyclin E2 overexpression is associated with endocrine resistance but not insensitivity to CDK2 inhibition in human breast cancer cells. Mol. Cancer Ther. *11*, 1488–1499. https://doi.org/10.1158/1535-7163.MCT-11-0963.

50. Hong, D.S., Kang, Y.K., Borad, M., Sachdev, J., Ejadi, S., Lim, H.Y., Brenner, A.J., Park, K., Lee, J.L., Kim, T.Y., et al. (2020). Phase 1 study of MRX34, a liposomal miR-34a mimic, in patients with advanced solid tumours. Br. J. Cancer *122*, 1630–1637. https://doi.org/10.1038/s41416-020-0802-1.

51. Franken, N.A.P., Rodermond, H.M., Stap, J., Haveman, J., and van Bree, C. (2006). Clonogenic assay of cells in vitro. Nat. Protoc. *1*, 2315–2319. https://doi.org/10.1038/nprot.2006.339.

52. Livak, K.J., and Schmittgen, T.D. (2001). Analysis of relative gene expression data using real-time quantitative PCR and the 2(-Delta Delta C(T)) Method. Methods *25*, 402–408. https://doi.org/10.1006/meth.2001.1262.

53. Cancer Genome Atlas Research Network; Analysis Working Group: Asan University; BC Cancer Agency; Brigham and Women's Hospital; Broad Institute; Brown University; Case Western Reserve University; Dana-Farber Cancer Institute; Duke University; Greater Poland Cancer Centre (2017). Integrated genomic characterization of oesophageal carcinoma. Nature *541*, 169–175. https://doi.org/10.1038/nature20805.

54. The Cancer Genome Atlas Research Network (2014). Comprehensive molecular characterization of gastric adenocarcinoma. Nature *513*, 202–209. https://doi.org/10.1038/nature13480.

55. Cerami, E., Gao, J., Dogrusoz, U., Gross, B.E., Sumer, S.O., Aksoy, B.A., Jacobsen, A., Byrne, C.J., Heuer, M.L., Larsson, E., et al. (2012). The cBio cancer genomics portal: an open platform for exploring multidimensional cancer genomics data. Cancer Discov. *2*, 401–404. https://doi.org/10.1158/2159-8290.CD-12-0095.

56. Harvard BIoMa (2016). Firehose, Broad Institute TCGA Genome Data Analysis Centre: 2016_01_28 run.

57. Mandard, A.M., Dalibard, F., Mandard, J.C., Marnay, J., Henry-Amar, M., Petiot, J.F., Roussel, A., Jacob, J.H., Segol, P., and Samama, G. (1994). Pathologic assessment of tumor regression after preoperative chemoradiotherapy of esophageal carcinoma. Clinicopathologic correlations. Cancer *73*, 2680–2686. https://doi.org/10.1002/1097-0142(19940601)73:11<2680::aid-cncr2820731105>3.0.co;2-c.

OMTN, Volume *37*

Supplemental information

miRNA-34a-5p is a novel biomarker and modulator of the radioresponse in esophageal adenocarcinoma

Christina Cahill, David Hackett, Laura E. Kane, James J. Phelan, Jason McGrath, Maitiú Ó. Murchú, Aoife Cannon, Aisling B. Heeran, Aoife Kilgallon, Rebecca Lyons, Rebecca M. O'Brien, Wei-Lin Winnie Wang, Meghana S. Menon, Oana M. Deac, Antonia Tierney, Narayanasamy Ravi, Claire Donohoe, Dermot O'Toole, Cian Muldoon, Aoife Maguire, Joanne Lysaght, Maeve A. Lowery, John V. Reynolds, Stephen G. Maher, Jacintha O'Sullivan, and Niamh Lynam-Lennon

Table S1: Cox regression multivariate analysis of prognostic ability

Parameter	HR	CI	*p* value
miR-34a expression<br>(low vs. high)	0.257	0.077-0.856	0.027 *
TRG<br>(TRG 1-2 vs. 3-5)	0.285	0.061-1.341	0.112
Tumour Size<br>(small vs. large)	0.492	0.173-1.397	0.183
Tumour differentiation<br>(poor + poor/moderate vs. moderate/well)	0.424	0.139-1.297	0.132
Pathological T stage<br>(T0-1 vs. T2-3)	3.892	1.069-14.171	0.039 *
Pathological N stage<br>(N0 vs. N1-2)	2.157	0.632-7.359	0.219

Abbreviations; HR, hazard ratio; CI, confidence interval; TRG, tumour regression grade; T stage, tumour stage; N stage, lymph node stage.

Table S2: Optimised clonogenic seeding densities for assessment of the radiosensitivity of OAC cells

Cell line	Treatment	Cells seeded per well (6-well plate)	Days incubated
OE19	0 Gy<br>2 Gy	1.5×10^3<br>3×10^3	~ 10
OE33	0 Gy<br>2 Gy	1.5×10^3<br>3×10^3	~ 7
OE33 P/R	0 Gy<br>2 Gy	1.5×10^3<br>3×10^3	~ 7
Flo-1^{Par}	0 Gy<br>2 Gy<br>4 Gy<br>6 Gy	1×10^3<br>2×10^3<br>4×10^3<br>6×10^3	~ 10
Flo-1^{LM}	0 Gy<br>2 Gy<br>4 Gy<br>6 Gy	1.5×10^3<br>3×10^3<br>6×10^3<br>1.2×10^4	~ 10

Abbreviations; Gy, Gray
